# PSNO: Predicting Cysteine *S*-Nitrosylation Sites by Incorporating Various Sequence-Derived Features into the General Form of Chou’s PseAAC

**DOI:** 10.3390/ijms150711204

**Published:** 2014-06-25

**Authors:** Jian Zhang, Xiaowei Zhao, Pingping Sun, Zhiqiang Ma

**Affiliations:** 1 School of Computer Science and Information Technology, Northeast Normal University, Changchun 130017, China; E-Mails: zhangj943@nenu.edu.cn (J.Z.); xwzhao_nenu@yeah.net (X.Z.); ppsun_nenu@yeah.net (P.S.); 2 National Engineering Laboratory for Druggable Gene and Protein Screening, Northeast Normal University, Changchun 130024, China

**Keywords:** cysteine *S*-nitrosylation sites, relative entropy selection, incremental feature selection, *k*-nearest neighbor

## Abstract

*S*-nitrosylation (SNO) is one of the most universal reversible post-translational modifications involved in many biological processes. Malfunction or dysregulation of SNO leads to a series of severe diseases, such as developmental abnormalities and various diseases. Therefore, the identification of SNO sites (SNOs) provides insights into disease progression and drug development. In this paper, a new bioinformatics tool, named PSNO, is proposed to identify SNOs from protein sequences. Firstly, we explore various promising sequence-derived discriminative features, including the evolutionary profile, the predicted secondary structure and the physicochemical properties. Secondly, rather than simply combining the features, which may bring about information redundancy and unwanted noise, we use the relative entropy selection and incremental feature selection approach to select the optimal feature subsets. Thirdly, we train our model by the technique of the *k*-nearest neighbor algorithm. Using both informative features and an elaborate feature selection scheme, our method, PSNO, achieves good prediction performance with a mean Mathews correlation coefficient (*MCC*) value of about 0.5119 on the training dataset using 10-fold cross-validation. These results indicate that PSNO can be used as a competitive predictor among the state-of-the-art SNOs prediction tools. A web-server, named PSNO, which implements the proposed method, is freely available at http://59.73.198.144:8088/PSNO/.

## 1. Introduction

*S*-nitrosylation (SNO) is one of the most ubiquitous post-translational modifications (PTMs) involving the covalent interaction of nitric oxide with the thiol group of cysteine residues [1]. Many lines of evidence have suggested that *S*-nitrosylation sites (SNOs) play key roles in providing proteins with structural and functional diversity, as well as in regulating cellular plasticity and dynamics. Malfunction or dysregulation of SNOs leads to a series of severe diseases [2], including developmental abnormalities and various diseases, such as cancer [3], Parkinson’s [4], Alzheimer’s [5] and amyotrophic lateral sclerosis [6]. Therefore, detecting possible SNO substrates and their corresponding exact sites is crucial for understanding the mechanisms of the biological processes of these diseases and promising great possibilities as effective therapeutic targets or diagnostic markers.

Several biochemical methodologies, including absorbance detection [7], colorimetric assays [8] and fluorescent assays [8,9], have been developed to identify SNOs. Compared with expensive and time-consuming biochemical experiments, computational methods are attracting more and more attention, due to their convenience and efficiency.

In 2001, Jaffrey made the first attempt to develop a biotin-switch technique (BST) for the large-scale detection of SNO substrates [10]. The BST includes three principal steps: (i) the methylthiolation of free protein thiols; (ii) the reduction of SNO bonds on Cys residues with ascorbate; and (iii) the ligation of thiols using *N*-[6-(Biotinamido)hexyl]-3'-(2'-pyridyldithio) propionamide (biotin-HPDP). Soon after that, Gross developed a predictor, named SNOSID [11]. This is a proteomic method, which identified endogenous and chemically-induced SNOs in proteins from tissues or cells. In 2009, Forrester explored a protein microarray-based approach using resin-assisted capture (RAC) to screen SNOs [12]. Compared with BST using a human embryonic kidney cell dataset, SNO-RAC outperformed it with higher sensitivity for proteins larger than ~100 kDa. Although these methods did make contributions to the development of the prediction of SNOs from different aspects, they were labor intensive and had a relatively low throughput.

Recent years have witnessed several computational methods that have been proposed in this field. Xue adopted a group-based prediction system for the prediction of kinase-specific SNOs and developed software named GPS-SNO (Group-based Prediction System) [13]. Li used a coupling pattern-based encoding scheme (CPR) and built a web server named CPR-SNO [14]. Xu introduced a position-specific amino acid propensity matrix to construct the predictor and built a free website, iSNO-pseudo-amino acid composition (PseAAC) [15]. As the iSNO-PseAAC treated all the proteins independently without taking into account any of their correlations, the following iSNO-AAPair incorporated some sequence correlation effects into the feature vector [16].

Each of the aforementioned methods has its own merit and does facilitate the development of this field. Although these computational models have been developed to predict SNOs, their accuracy is unsatisfactory, and they lack a detailed analysis of the features. Therefore, it is important to develop an efficient method for the site-specific detection of SNOs.

In this paper, we focus on the challenging problem of predicting SNOs based on primary sequence information. A novel method, PSNO, is proposed for differentiating SNOs from non-SNOs. Firstly, various informative sequence-derived features that effectively reflect the intrinsic characters of a given peptide are combined to construct informative features; Secondly, relative entropy selection and incremental feature selection are adopted to select the optimal feature subsets; Thirdly, we use *k*-nearest neighbor to identify SNOs based on the selected optimal feature subsets. In order to evaluate the proposed method with previous works fairly, 10-fold cross-validation is implemented on the widely-used low-similarity training dataset. The experimental results show that the proposed PSNO is a powerful computational tool for SNOs prediction. A web-server, named PSNO, that implements the proposed method is freely available at http://59.73.198.144:8088/PSNO/.

## 2. Results and Discussion

### 2.1. The Feature Selection Results

The output of the relative entropy selector was two lists: one was called the feature list, which sorted the features according to their importance to the class of samples; the other was called the coefficient list, which sorted the coefficient values in descending order (Table S1). In the coefficient value list, a feature with a larger index implied that it tended to play a more important role in identifying SNOs. Such a list of ranked features would be used in the following IFS procedure for searching the optimal feature subset.

**Figure 1:**
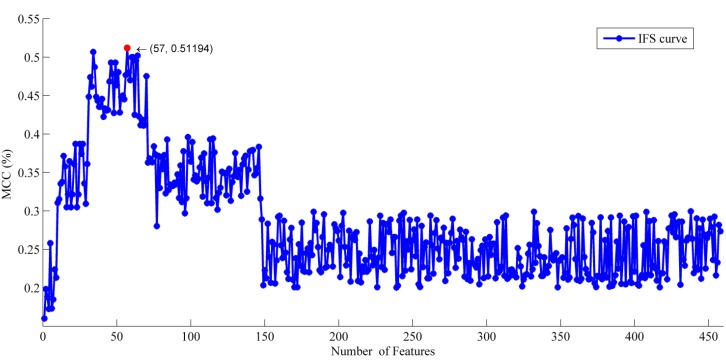
The IFS curve of 458 features for the training dataset. The *x*-axis and *y*-axis indicatesthe mean Mathews correlation coefficient (*MCC*) and number of features, respectively. When the number of selected features is 57, the mean *MCC* reaches the maximum, 0.51194.

Based on the results of the relative entropy selector, 458 individual classifiers were built by adding features one by one from the top of the feature list to the bottom (Table S2). As shown in Figure 1, the mean *MCC* values reached the maximum when 57 features were provided.

In this paper, 10-fold cross-validation was performed on the training dataset (731 SNOs and 810 non-SNOs). We obtained a mean accuracy of 68.85% using all the features with a sensitivity of 67.99%, a specificity of 69.63% and an *MCC* of 0.3759. Using 57 optimal features, our model produced 75.67% accuracy with 74.15% sensitivity, 77.04% specificity and an *MCC* of 0.5119. The results suggested that our feature selection approach successfully chose “good” features, as well as eliminated “bad” features.

### 2.2. Analysis of the Optimal Feature Set

**Figure 2:**
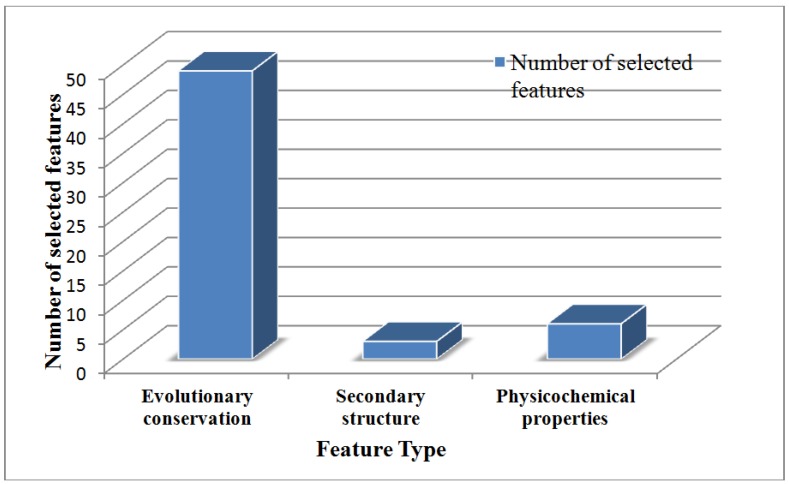
The distribution of each feature type in the final optimal feature subset. The *x*-axis and *y*-axis indicate the feature type and the number of selected features, respectively. Of the 57 optimal features, 48 belong to the evolutionary conservation score, three to the predicted secondary structure and six to the physicochemical properties.

To discover the different contributions of various types of features, we further investigated the distribution of each kind of feature in the final optimal feature subset. The results are shown in Figure 2. Of the 57 optimal features, 48 belonged to the evolutionary conservation score, three to the predicted secondary structure, six to the physicochemical properties, which indicated that all three types of features contribute to the prediction of protein SNOs. The detailed descriptions of the 57 optimal features are shown in the Table S3. In addition, evolutionary conservation scores accounted for the biggest part in differentiating SNOs from non-SNOs.

As is well known, all biological species were developed starting from a very limited number of ancestral species. Evolution was an eternal process that impenetrated the whole history of life. The evolution of protein sequences involved the changes, insertions and deletions of single residues or peptides along with the entire development of proteins [17]. Although some similarities may be eliminated after a long time of evolution, the corresponding protein zones may still share some common attributes, because the functional sites of a protein always locate in the conservation zone [18]. This explains why evolutionary conservation scores occupy the biggest part of the optimal subset. In addition, the features within the top 10 features in the final optimal feature subsets contained seven evolutional profile features.

**Figure 3:**
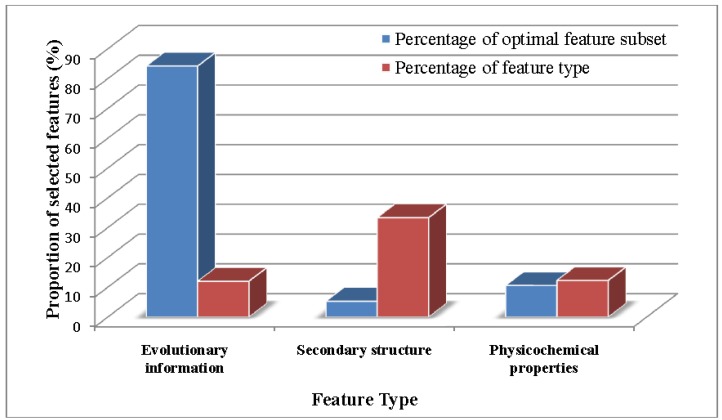
The proportion of each type of feature in the optimal feature subset. The *x*-axis and *y*-axis indicate the feature type and the proportion of the selected features, respectively. The blue blocks represent the percentage of the selected features accounting for the whole optimal feature subsets, and the red ones represent the percentage of the selected features accounting for the corresponding feature type.

We also calculated different kinds of features accounting for the various proportions of the optimal feature subset (Figure 3). The blue blocks represented the percentage of the selected features accounting for the whole optimal feature subsets, and the red ones represented the percentage of the selected features accounting for the corresponding feature type. Although, within the final optimal feature subset, a few secondary structure features are selected, we cannot say that the secondary structure features are not tightly related to SNOs. Among all nine secondary structure features, three features were selected in the optimal feature subsets.

### 2.3. Comparison of PSNO with Other Methods

In this section, we compare PSNO with GPS-SNO [13], iSNO-PseAAC [15] and iSNO-AAPair [16], which were all sequence-based prediction methods. As the iSNO-AAPair was built on a different dataset (1530 human and mouse proteins), we adopted the independent dataset to compare our PSNO with iSNO-AAPair. In order to reach a consensus assessment with GPS-SNO and iSNO-PseAAC, a 10-fold cross-validation was adopted here to examine the prediction quality. Listed in Table 1 are the corresponding results obtained by the aforementioned two methods on the same training dataset. As can be seen, the *SN*, *ACC* and *MCC* rates achieved by PSNO were obviously higher than those by GPS-SNO with different thresholds and iSNO-PseAAC. Although the GPS-SNO ^1^ achieved the highest *SP* value, the *SN* and *MCC* value was relatively low. It may be that when the threshold parameter was set at “high”, more non-SNOs tended to be correctly classified, while some SNOs were mistakenly identified as non-SNOs.

Listed in the Table S4 are the predicted results by PSNO for Xue’s independent dataset. As we can see from Table S4, of the 2302 SNOs, 2188 were successfully identified. The overall success rate was about 95.05%.

In order to assess the ability of the proposed PSNO for practical applications, we adopted Xu’s independent dataset containing 81 SNO and 100 non-SNO experimentally-verified peptides. Among the existing models for the prediction of the SNOs, the web server for the model proposed in [14] did not work, and the method in [19] had no web server at all. Therefore, the comparison was made among the following four methods: GPS-SNO, iSNO-PseAAC, iSNO-AAPair and ours, PSNO. Table 2 summarizes the results of PSNO with the existing prediction methods for the four different metrics. Using the optimal 57 features, the *SN*, *SP*, *ACC* and *MCC* values produced by PSNO are 87.7%, 85.0%, 86.2% and 0.72, respectively, which are about 8.1%~43.2%, 0.9%~9.8%, 5.5%~24.6% and 0.09~0.44 higher than previous studies.

**Table 1:** The performance comparison of PSNO with other existing prediction methods on the training dataset. GPS, group-based prediction system.

Predictor	*SN* (%)	*SP* (%)	*ACC* (%)	*MCC*
GPS-SNO ^1^	18.88	**89.63**	56.07	0.1210
GPS-SNO ^2^	28.04	81.98	56.39	0.1193
GPS-SNO ^3^	45.01	73.33	59.90	0.1915
iSNO-PseAAC	67.01	68.15	67.62	0.3515
PSNO	**74.15**	77.04	**75.67**	**0.5119**

^1^ The method proposed in [13] where the threshold parameter was set at “high”; ^2^ the method proposed in [13] where the threshold parameter was set at “medium”; ^3^ the method proposed in [13] where the threshold was set at “low”. *SN*, *SP*, *ACC* and *MCC* represented the sensitivity, specificity, accuracy and the Mathews correlation coefficient, respectively.

**Table 2:** Comparison of PSNO with the existing prediction methods via Xu’s independent dataset.

Predictor	*SN* (%)	*SP* (%)	*ACC* (%)	*MCC*
GPS-SNO ^1^	44.5	81.0	64.7	0.28
iSNO-PseAAC	50.2	75.2	62.8	0.30
iSNO-AAPair	79.6	84.1	81.7	0.63
PSNO	**87.7**	**85.0**	**86.2**	**0.72**

^1^ The method proposed in [13] where the threshold parameter was set at “medium”. *SN*, *SP*, *ACC* and *MCC* represented the sensitivity, specificity, accuracy and the Mathews correlation coefficient, respectively.

In practical applications, the input should be entire protein sequences. To test the state-of-the-art web servers used for practical applications, our independent dataset (see Section 3.1) was used here. The predicted results are shown in Table 3. Our PSNO produced an *MCC* of 0.4475, which was about 14.22%~32.29% higher than previous studies.

**Table 3:** Comparison of PSNO with the existing prediction methods using our independent dataset. PseAAC, pseudo-amino acid composition.

Predictor	*SN* (%)	*SP* (%)	*ACC* (%)	*MCC*
GPS-SNO ^1^	41.51	**70.87**	60.90	0.1244
iSNO-PseAAC	60.38	67.96	65.38	0.2722
iSNO-AAPair	66.04	66.02	66.03	0.3053
PSNO	**79.25**	67.96	**71.79**	**0.4475**

^1^ The method proposed in [13] where the threshold parameter was set at “medium”. SN, SP, ACC and MCC represented the sensitivity, specificity, accuracy and the Mathews correlation coefficient, respectively.

### 2.4. Implementation of PSNO Server

For the convenience of biology scientists, PSNO has been implemented as a free web server located at http://59.73.198.144:8088/PSNO/. Here, a step-by-step brief guide is given below to describe how to use it.

**Figure 4:**
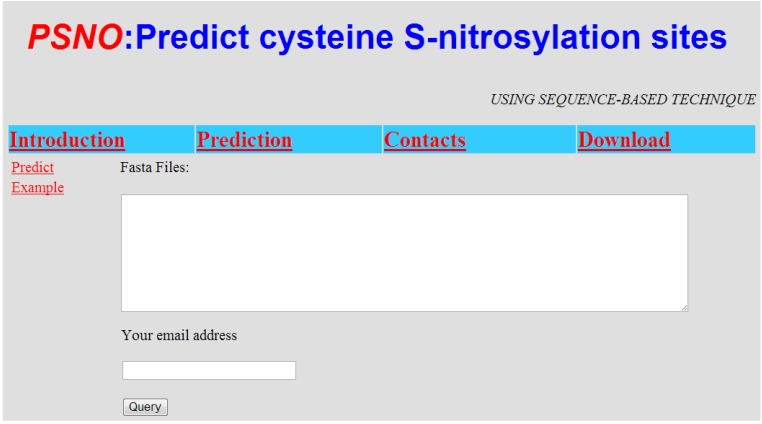
The home page of the PSNO web server.

Step 1. Access the web server, and the home page is the default interface displayed (Figure 4). Click on the “Introduction” link to see a detailed description about the server, which includes the User’s Guide, “Input”, “Output”, “Limitation” and “Requirement”.

Step 2. You can either type or paste the query sequence into the text box in Figure 4. The query sequence should be in the FASTA format. The FASTA format sequence consists of a single initial line beginning with a symbol (“>”), followed by lines of sequence data. You can click on the “Example” link to see the example sequences. You are also required to provide a valid email address in the text box.

Step 3. Click on the “Query” button to submit the computation request. PSNO begins processing and the predicted probabilities of a site being an SNOs or non-SNOs will be sent to you through the email provided.

## 3. Materials and Methods

### 3.1. Benchmark Datasets

In order to reach a consensus assessment with previous studies [13,15,16], four datasets were used in this paper. The training dataset used in this paper was derived from dbSNO (http://dbsno.mbc.nctu.edu.tw), which integrated the experimentally verified cysteine SNOs from different species [20]. The training dataset contained 731 experimentally-verified SNOs and 810 experimentally verified non-SNOs from 438 randomly selected proteins, none of which had more than 40% similarity to any other. The peptide segments for SNOs and non-SNOs could be formulated by:

*P* = *R*_−*ξ*_*R*−_(*ξ*−1)_…*R*_−2_*R*_−1_*CR*_+1_*R*_+2_…*R*_+(*ξ*−1)_*R*_+*ξ*_ (1)

where *R*_−ξ_ and *R*_ξ_ represented the ξ-th downstream and upstream residues from cysteine (C), respectively. *P* represented the peptide being either an SNO peptide or a non-SNO peptide. To test our method, as well as to reach a consensus assessment with previous investigators [13,15,16], ξ was set as 10 to compile the training dataset. If the upstream or downstream for a cysteine was less than 10, the lacking residues would be filled with dummy code X.

Xue’s independent dataset [13,15] consisted of 461 experimentally-verified nitrosylated proteins from published literature or the UniProt database (http://www.uniprot.org/). All of these proteins are clustered with a threshold of less than 40% identity by CD-HIT (Cluster Database at High Identity with Tolerance) [21]. After using the same technique mentioned above, 2302 SNOs are compiled from the 461 nitrosylated proteins. None of these 2302 SNOs occurred in the training dataset. In [16], Xu developed a public independent dataset (81 SNOs and 100 non-SNOs). The corresponding nitrosylated proteins and sequences were taken from dbSNO and UniProt, respectively.

In practical applications, the input should be entire protein sequences. To test the state-of-the-art web servers used for practical applications, we collected a new independent dataset by extracting the experimental-verified 20 nitrosylated proteins from dbSNO. None of them occurred in the training dataset. After compiling based on the same technique, 53 SNOs and 103 non-SNOs are obtained from the 20 nitrosylated proteins. The sequences of these 20 proteins, as well as SNOs (red) and non-SNOs (blue) are freely available at our PSNO web server. Table 4 summarizes the detailed compositions of above-mentioned four datasets.

**Table 4:** Detailed compositions of the four datasets.

Dataset	Proteins	Peptides	SNOs	Non-SNOs
Training dataset	438	1541	731	810
Xue’s independent dataset	461	2302	2302	0
Xu’s independent dataset	-	181	81	100
Our independent dataset	20	156	53	103

“-” The paper [16] makes no mention.

### 3.2. Sample Formulation and Feature Construction

In order to build a powerful protein system, the first thing was to represent the sequences with proper and effective mathematical expressions, which can reflect the intrinsic correction with the target to be predicted. In this study, we incorporated sequence-derived features into pseudo-amino acid composition (PseAAC) to represent the sample of a target protein. The PseAAC method had been widely used in bioinformatics, such as identifying proteins attributes [22,23], predicting protein structures [24,25] and predicting protein classes [26,27]. According to a recent review [28], the general form of PseAAC for a protein could be formulated as:

*P* = [*ψ*_1_, *ψ*_2_ …, *ψ*_μ_, …, *ψ*_Ω_]^*T*^ (2)

where *T* was a transpose operator and the *ψ*_1_, *ψ*_2_ … depended on how to extract the desired information from the protein sequence of *P*. Here, several sequence-derived features were explored to distinguish the SNOs and non-SNOs. These features included evolutionary conservation scores, the predicted secondary structure and physicochemical properties.

#### 3.2.1. Features of Evolutionary Conservation Scores

Evolutionary conservation scores had been widely used by the investigators to predict various attributes of proteins, such as predicting the protein subcellular location [29], identifying the subnuclear protein location [30] and identifying the protease family [31]. To incorporate evolutionary conservation scores, PSSM (Position-specific Scoring Matrix) was generated by the program “blastpgp” (PSIBLAST) [32], which was used to search the Swiss-Prot database (released on 15 May 2011; http://www.ebi.ac.uk/swissprot/) through 3 iterations (−j 3) and an *e*-value threshold of 0.0001 (−h 0.0001) for multiple sequence alignment against the protein, *P*. According to [33], the sequence evolution information of protein *P* with *L* amino acid residues could be expressed by a 20 × *L* matrix, as given by:

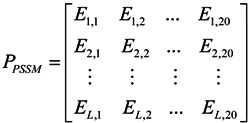
(3)
where *E_i,j_* represented the score of the amino acid in the *i-*th position of the sequence that was being changed to amino acid type *j* (*j* = 1, 2, …, 20) during the evolutionary process.

PSSM scores were generally displayed as positive or negative integers. Positive scores (ratio > 0) indicated that the given amino acid substitution exceeded the expected frequency, suggesting that this substitution was surprisingly favored in the alignment than expected by chance, while negative scores (ratio < 0) indicated the opposite; that the frequency occurred less than the expected frequency, suggesting that the substitution was not favored. 

**Figure 5:**
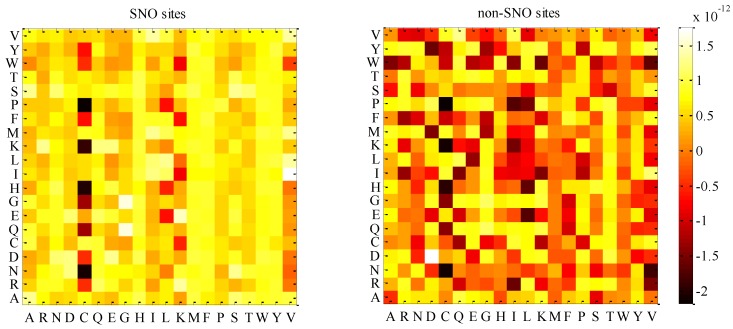
The heat maps of the preference of evolutionary conservation in *S*-nitrosylation sites (SNOs) and non-SNOs.The yellow color indicates the higher probability of appearance of evolutionary conservation, while the black color indicates less appearance.

The preference of evolutionary conservation in SNOs and non-SNOs were calculated and displayed in a heat map (Figure 5). In this figure, amino acids were sorted in both the *x*-axis and *y*-axis. The color palette from black to yellow indicated a growing preference for evolutionary conservation in SNOs and non-SNOs. The yellow color indicated the higher probability of the appearance of evolutionary conservation, while the black color meant less appearance. For instance, the substitution of C/H (*x*-axis/*y*-axis) was black, while the H/C (*x*-axis/*y*-axis) was yellow in SNOs, which suggested that the mean probabilities (or tendency) for His being substituted by Cys was higher than that for Cys being substituted by His in the SNOs. In addition, the H/C of non-SNOs was red. This determined the mean probabilities (or tendency) for His being substituted by Cys in SNOs being higher than those in non-SNOs. Generally speaking, compared with non-SNOs, evolutionary-conserved sets were preferred to aggregate in SNOs, which indicated critical active sites or functional residues that may be required for other intermolecular interactions being abundant in these peptides.

In order to make the descriptor uniformly cover the peptide, we used the elements in the above equation for PSSM (Equation (3)) to define a new matrix, *M_PSSM_*, as formulated by:

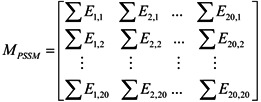
(4)
where the value of ∑*E_i,j_* equaled the sum of amino acid type *i* being changed to amino acid type *j* in above-mentioned matrix *P_PSSM_*. In summary, 400 features were obtained to construct features of evolutionary conservation scores.

#### 3.2.2. Features of Predicted Secondary Structure

Consider the fact that proteins with low sequence similarity, but in the same structural class, are likely to share high similarity in their corresponding secondary structural elements. Therefore, it would be useful to encode the protein sequences by taking into account the secondary structure information. In this study, several predicted secondary structure-based features were introduced to further improve low-similarity protein prediction accuracy. In this work, PSIPRED [34] was adopted to explore the secondary structure of a query protein sequence. The outputs of PSIPRED were encoded in terms of “C” for coil, “H” for helix and “E” for strand. The total number, average length and composition percent of C, H and E segments were calculated and constructed for the predicted secondary structure features. These features were defined as follows:

*Total_number_α_* = *∑α* (5)

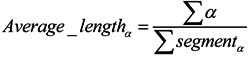
(6)

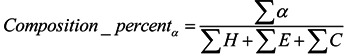
(7)
where α = {*H*, *E*, *C*}, *∑α* is the sum of the secondary structure of type α in the peptide. *∑segment_α_* is the sum of segments of type α in the peptide. As a result, 3 + 3 + 3 = 9 features were obtained to construct the predicted secondary structure features.

#### 3.2.3. Features of Physicochemical Properties

Forty nine selected physical chemical, energetic and conformational properties, which have been observed to be widely used in pre-works [29,35,36], were used here. More detailed descriptions can be found at http://www.cbrc.jp/~gromiha/fold_rate/property.html. For each sequence, 49 properties values were firstly calculated by taking the sum of each property value over the whole residues and then divided by the length of the sequence. In this encoding scheme, a peptide was encoded by a 49-dimensional vector.

### 3.3. The Relative Entropy Selection

Commonly, the combination of various features would bring more informative features to the classifier. Nevertheless, some “bad” features were also added and became the unwanted noise. This noise, which was redundant with other features, may deteriorate the performance of learning algorithms and decrease the generalization power of the learned classifiers [37]. In order to get rid of the related or noisy feature, the feature selection approach for the optimal subset of features from a high-dimensional feature space was a critical job in machine learning. Relative entropy selection (*i.e.*, Kullback–Leibler divergence) [38] was proven to be a powerful method to identify those features that were the most useful in describing the essential differences among the possible classes. In this algorithm, relative entropy can be defined the as:

*D_KL_(P||Q) + D_KL_(Q||P)* (8)

where *P* and *Q* are the conditional probability density function of a feature under two different classes; *D_KL_(P||Q)* is the *K*–*L* divergence of *Q* from *P* and *D_KL_(Q||P)* was the *K*–*L* divergence of *P* from *Q*. After the calculation, we got a feature list, *L*:

*L* = {*f*_1_, *f*_2_, *f*_3_,…,*f_i_*,…} and *i* = {1,2,3…*N*} (9)

In this feature list, *L*, the index, *i*, of each feature indicated the importance of *f_i_* to the class of the sample.

### 3.4. Incremental Feature Selection

Through the relative entropy selection, we obtained the ranked feature list. In order to determine which features should be selected for the optimal feature set for our model, the incremental feature selection (IFS) procedure [19] was adopted here to search for a good feature subset involving finding those features that were highly correlated with the decision features, but that are uncorrelated with each other.

During the IFS procedure, we added the feature in the ranked feature list one by one from the top to the bottom. After a feature had been added, a new feature subset was composed. For each new feature subset, a classifier was built based on the new feature subset using 10-fold cross-validation on the training dataset. As a result, 458 individual classifiers were constructed for the 458 feature subsets. By doing so, a table named IFS, with one column for the feature index and the other column for the prediction performance of each individual classifier, was obtained. An IFS curve was drawn to identify the best prediction performance, as well as the corresponding optimal feature subsets.

### 3.5. K-Nearest Neighbor Algorithm

The *k*-nearest neighbor algorithm (KNN) is quite popular in pattern recognition and machine learning. According to the KNN algorithm [39], the query sample would be assigned to the subset represented by its *k*-nearest neighbors. In this study, if the majority of the *k*-nearest neighbors of the query sample is a positive sample, this means that it is an SNO site. Otherwise, the query sample is regarded as a negative one. There are many different distances to measure the nearest neighbors for the KNN algorithm, such as the Hamming distance [40], Euclidean distance [40] and the Mahalanobis distance [41]. In order to build a KNN model, we tested different *k*-values from 3 to 19, as well as various different definitions. The best performance was achieved with *K* = 9 using the Euclidean distance. 

### 3.6. Assessment of Prediction Accuracy

Four routinely used evaluation indexes were adopted in this paper, *i.e.*, sensitivity (*SN*), specificity (*SP*), accuracy (*ACC*) and the Mathews correlation coefficient (*MCC*).

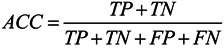
(10)

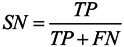
(11)

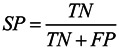
(12)

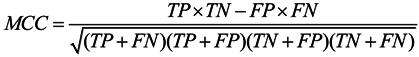
(13)
where *TP*, *TN*, *FP* and *FN* were the abbreviations of true positives, true negatives, false positives and false negatives. In this paper, *MCC* was used as the major evaluation criteria to evaluate the performance of the proposed approach as the positive and negative samples in the training dataset were imbalanced.

### 3.7. Cross-Validation Test

**Figure 6:**
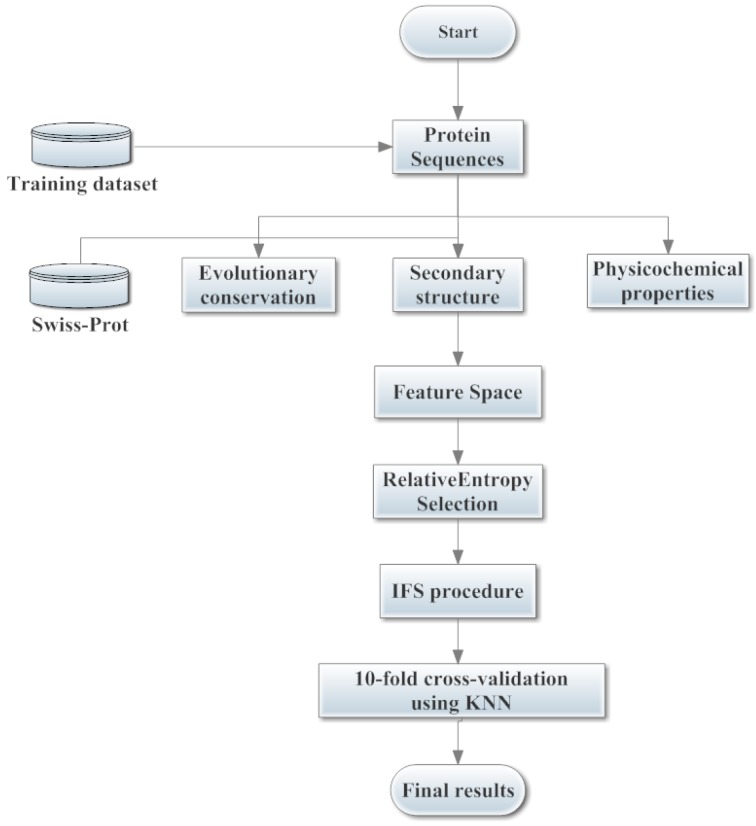
The system architecture of the proposed model. Three different types of sequence-derived features, *i.e.*, evolutionary conservation, secondary structure and physicochemical properties, are generated and constructed as the feature space. Relative entropy selection and the incremental feature selection (IFS) procedure are adopted to select the optimal feature subset. The final results are obtained by using 10-fold cross-validation based on the *k*-nearest neighbor (KNN) and the selected optimal feature subsets.

In statistical prediction, the independent dataset, sub-sampling (*k*-fold cross-validation) and jackknife analysis (leave-one-out) are the three cross-validation methods that are often used to assess a prediction tool for its effectiveness in practical application. In order to reach a consensus assessment with previous studies [13,15,16], we used the same 10-fold cross-validation to examine the prediction performance as done by many studies for SNOs prediction. Firstly, the dataset was randomly divided into ten equal subsets; then, nine subsets were used for training and the remaining one for testing. The procedure was repeated 10 times, and the final performance was calculated by averaging over 10 testing sets. The system architecture of the proposed model is illustrated in Figure 6.

## 4. Conclusions

In this paper, we present a novel method named PSNO based on sequence-derived features and effective feature selection techniques to identify SNOs. The PSNO model achieves a promising performance and outperforms many other prediction tools. We ascribe the excellent performance of our predictor PSNO to two aspects. The first aspect is the informativeness of the feature vector in our model in representing proteins. The feature vector in this study includes an evolutionary profile, a secondary structure and physicochemical properties. However, rich information also brings the enlargement of the dimension and worsening of the predictor, which needs a proper feature selection strategy. Therefore, the second aspect is the effectiveness of relative entropy selection, followed by the IFS procedure. By means of powerful feature selection, an optimal set of 57 features, which contribute significantly to the prediction of SNOs, are selected. With the 57 optimal features selected, our predictor achieves an overall accuracy of 75.67% and an *MCC* of 0.5119 on a training dataset using 10-fold cross-validation. Theoretically, the protein structures can bring rich information to construct powerful prediction models compared to simple sequences. However, the sequence-based prediction is an alternative to the structure-based prediction in the absence of structures. As a result of the completion of whole-genome sequencing projects, the sequence-structure gap is rapidly increasing. Thus, it would be a powerful prediction tool to identify SNOs for newfound proteins without structure information. For the convenience of biology scientists, the proposed PSNO has been implemented as a web server and is freely available.

## Supplementary Files

Supplementary File 1
