# Supplementary material for: PSNO: Predicting Cysteine S-Nitrosylation Sites by Incorporating Various Sequence-Derived Features into the General Form of Chou’s PseAAC
Source: Int J Mol Sci. 2014 Jun 25;15(7):11204–19. doi: 10.3390/ijms150711204 (PMC4139777; doi:10.3390/ijms150711204)
Supplement: Supplementary File 1 [file ijms-15-11204-s001.pdf]

Supplementary Information

Table S1. The first column is the row number; the second is the feature index; The third is the coefficient list. The features are sorted in descending order according to the coefficient list; The larger the value of the coefficient the more important its contribution.

No.	Feature Index	Coefficient List
1	440	37.683
2	295	31.312
3	146	27.184
4	148	27.184
5	416	23.222
6	442	22.634
7	85	20.361
8	64	19.271
9	127	18.371
10	22	17.177
11	274	16.812
12	211	16.486
13	204	16.077
14	206	16.071
15	208	16.071
16	436	16
17	203	15.385
18	379	15.222
19	1	14.806
20	169	14.614
21	441	13.809
22	358	13.738
23	232	13.513
24	43	12.654
25	150	12.131
26	151	12.114
27	413	11.635
28	337	11.48
29	402	11.299
30	125	11.122
31	78	10.818
32	316	10.54
33	400	9.7708
34	190	9.3035
35	215	9.1337
36	209	9.133
37	405	9.116
38	207	9.0315
39	298	9.0213

Table S1. *Cont.*

No.	Feature Index	Coefficient List
40	294	8.9584
41	212	8.6726
42	216	8.6429
43	160	8.5049
44	186	8.3461
45	188	8.3461
46	386	8.186
47	388	8.186
48	384	8.1018
49	71	8.0502
50	134	8.0344
51	130	7.9558
52	154	7.8462
53	18	7.737
54	401	7.7345
55	389	7.591
56	159	7.2968
57	65	7.2837
58	74	7.2756
59	189	7.2494
60	253	7.0844
61	25	7.0451
62	183	7.0329
63	153	6.7832
64	187	6.7606
65	184	6.7429
66	138	6.464
67	131	6.3728
68	318	6.2627
69	234	6.1283
70	275	6.0066
71	264	5.9585
72	70	5.9531
73	80	5.9312
74	158	5.8915
75	225	5.8437
76	145	5.7555
77	58	5.7404
78	390	5.6781
79	383	5.6507
80	238	5.6417
81	398	5.6198
82	230	5.5688
83	382	5.2796

Table S1. *Cont.*

No.	Feature Index	Coefficient List
84	291	5.1801
85	299	5.1604
86	285	5.123
87	290	5.1119
88	267	4.9686
89	38	4.934
90	14	4.9014
91	30	4.8917
92	387	4.8906
93	34	4.8604
94	142	4.8567
95	395	4.814
96	200	4.7975
97	51	4.789
98	202	4.7873
99	195	4.7663
100	404	4.7388
101	182	4.7337
102	79	4.7267
103	73	4.7223
104	192	4.6911
105	198	4.6673
106	231	4.6137
107	50	4.6096
108	40	4.5962
109	392	4.5854
110	114	4.5618
111	140	4.4376
112	311	4.3727
113	314	4.3292
114	213	4.2325
115	240	4.2229
116	310	4.193
117	266	4.1513
118	268	4.1513
119	218	4.1396
120	210	4.0842
121	403	3.9738
122	272	3.9155
123	201	3.9146
124	448	3.9015
125	293	3.8755
126	196	3.7635
127	105	3.7451

Table S1. *Cont.*

No.	Feature Index	Coefficient List
128	110	3.7124
129	279	3.708
130	84	3.6254
131	87	3.613
132	82	3.5772
133	19	3.5749
134	338	3.5577
135	26	3.5359
136	28	3.5359
137	428	3.5209
138	263	3.5114
139	9	3.4883
140	4	3.4647
141	262	3.379
142	3	3.3592
143	54	3.3384
144	60	3.197
145	45	3.1702
146	265	3.127
147	155	3.1244
148	149	3.0824
149	364	3.0656
150	222	3.0582
151	276	3.0572
152	170	3.0426
153	374	3.0075
154	147	2.9995
155	157	2.9389
156	152	2.9214
157	139	2.9208
158	133	2.8884
159	11	2.864
160	126	2.8523
161	128	2.8523
162	366	2.8461
163	368	2.8461
164	375	2.8335
165	300	2.8153
166	319	2.7912
167	124	2.741
168	320	2.6502
169	2	2.6348
170	180	2.582

Table S1. *Cont.*

No.	Feature Index	Coefficient List
171	406	2.5283
172	396	2.5135
173	32	2.504
174	239	2.4879
175	226	2.4533
176	228	2.4533
177	289	2.3594
178	344	2.341
179	31	2.3353
180	286	2.3336
181	288	2.3336
182	244	2.3146
183	282	2.3112
184	365	2.2881
185	283	2.2819
186	35	2.2267
187	86	2.1964
188	88	2.1964
189	67	2.1918
190	334	2.1907
191	92	2.179
192	89	2.1745
193	39	2.1661
194	99	2.1366
195	165	2.1326
196	94	2.0666
197	83	2.0641
198	339	2.0418
199	24	2.0412
200	95	2.0307
201	98	2.0278
202	171	2.0138
203	191	2.0066
204	246	1.9993
205	248	1.9993
206	329	1.9885
207	61	1.9864
208	261	1.978
209	62	1.9379
210	326	1.9161
211	328	1.9161
212	313	1.8346
213	10	1.8345

Table S1. *Cont.*

No.	Feature Index	Coefficient List
214	120	1.8303
215	255	1.7995
216	419	1.7635
217	118	1.7591
218	111	1.7402
219	178	1.7311
220	233	1.716
221	355	1.6997
222	343	1.6918
223	75	1.6847
224	106	1.6711
225	108	1.6711
226	317	1.6594
227	107	1.6521
228	284	1.6179
229	277	1.5419
230	53	1.5363
231	59	1.5191
232	55	1.4649
233	21	1.419
234	33	1.4155
235	41	1.34
236	378	1.3298
237	363	1.3248
238	367	1.3118
239	369	1.2704
240	372	1.27
241	336	1.2421
242	144	1.2401
243	362	1.2271
244	361	1.2235
245	376	1.2148
246	377	1.2132
247	16	1.1492
248	220	1.1125
249	205	1.1124
250	15	1.0965
251	219	1.0849
252	217	1.049
253	175	1.0375
254	119	1.0319
255	243	1.0204
256	179	1.014

Table S1. *Cont.*

No.	Feature Index	Coefficient List
257	93	0.99941
258	352	0.9677
259	166	0.96565
260	168	0.96565
261	347	0.96454
262	356	0.96448
263	342	0.96436
264	305	0.96003
265	13	0.95219
266	247	0.94958
267	360	0.94935
268	7	0.94756
269	341	0.94696
270	251	0.94179
271	350	0.93945
272	346	0.92314
273	348	0.92314
274	303	0.91757
275	199	0.91561
276	249	0.91239
277	12	0.90343
278	161	0.88682
279	177	0.88299
280	173	0.87755
281	301	0.85791
282	315	0.85412
283	181	0.85411
284	357	0.83406
285	325	0.78883
286	345	0.78406
287	349	0.77777
288	256	0.77229
289	66	0.76238
290	68	0.76238
291	135	0.76112
292	391	0.73843
293	258	0.73804
294	393	0.72802
295	132	0.72493
296	235	0.72311
297	121	0.71383
298	309	0.71165
299	302	0.71137

Table S1. *Cont.*

No.	Feature Index	Coefficient List
300	399	0.71106
301	331	0.7059
302	137	0.70497
303	227	0.69879
304	242	0.67621
305	245	0.67447
306	252	0.67393
307	394	0.66355
308	385	0.66268
309	193	0.65964
310	63	0.64536
311	237	0.64392
312	330	0.63143
313	335	0.62971
314	221	0.62904
315	418	0.62709
316	229	0.62601
317	224	0.62351
318	115	0.6115
319	287	0.60671
320	324	0.60316
321	296	0.60095
322	185	0.59958
323	292	0.59762
324	370	0.59454
325	417	0.59366
326	297	0.5853
327	44	0.58259
328	322	0.57899
329	281	0.57204
330	37	0.5644
331	197	0.55697
332	332	0.54806
333	69	0.5469
334	327	0.54162
335	36	0.5327
336	333	0.52386
337	77	0.52368
338	112	0.51359
339	102	0.50815
340	72	0.49645
341	49	0.4816
342	278	0.47371
343	359	0.46675

Table S1. *Cont.*

No.	Feature Index	Coefficient List
344	56	0.4588
345	269	0.44195
346	101	0.42798
347	351	0.42116
348	117	0.41778
349	96	0.38954
350	280	0.37999
351	97	0.36473
352	410	0.35083
353	380	0.34818
354	444	0.34266
355	109	0.34262
356	353	0.33769
357	174	0.3344
358	90	0.33352
359	371	0.31886
360	164	0.31689
361	91	0.31586
362	100	0.3146
363	373	0.31261
364	250	0.28729
365	407	0.27357
366	260	0.25759
367	163	0.25346
368	172	0.2381
369	176	0.2369
370	5	0.23213
371	408	0.23104
372	259	0.2274
373	450	0.22491
374	257	0.22488
375	29	0.22079
376	241	0.22015
377	6	0.2007
378	8	0.2007
379	17	0.19509
380	42	0.18596
381	323	0.18221
382	214	0.18181
383	381	0.1765
384	306	0.17617
385	308	0.17617
386	23	0.17246
387	304	0.16167

Table S1. *Cont.*

No.	Feature Index	Coefficient List
388	20	0.15814
389	321	0.15114
390	446	0.14947
391	409	0.14873
392	340	0.12959
393	455	0.12948
394	143	0.12254
395	453	0.11209
396	81	0.1091
397	354	0.10849
398	46	0.10742
399	48	0.10742
400	141	0.0985
401	449	0.098495
402	451	0.095382
403	443	0.094035
404	57	0.093523
405	162	0.091505
406	223	0.091191
407	113	0.084302
408	445	0.082408
409	129	0.079377
410	194	0.078343
411	271	0.076524
412	307	0.068983
413	431	0.064972
414	414	0.064581
415	454	0.064571
416	312	0.060771
417	156	0.056122
418	397	0.054426
419	457	0.053581
420	122	0.052359
421	52	0.051213
422	456	0.05097
423	123	0.050425
424	104	0.047095
425	103	0.046691
426	47	0.045374
427	27	0.043113
428	236	0.041121
429	270	0.037554
430	167	0.037316
431	273	0.037281

Table S1. *Cont.*

No.	Feature Index	Coefficient List
432	458	0.034997
433	412	0.032788
434	447	0.030807
435	439	0.027213
436	411	0.024556
437	116	0.024037
438	420	0.021777
439	136	0.017523
440	432	0.016289
441	438	0.015336
442	76	0.014952
443	254	0.010339
444	452	0.008206
445	433	0.007503
446	434	0.007219
447	435	0.006495
448	426	0.003572
449	425	0.002848
450	427	0.002534
451	424	0.002518
452	437	0.001999
453	415	0.001629
454	423	0.000925
455	430	0.000596
456	429	0.000498
457	421	0.000422
458	422	0.000373

Table S2. Based on the results of Relative Entropy Selector, 458 individual classifiers were built by adding features one by one from the top of the feature list to the bottom. The mean MCC values reached the maximum when 57 features were provided (See the following table in red).

Number of Features	*MCC* Value
1	0.16
2	0.198307
3	0.188261
4	0.1524
5	0.25799
6	0.162991
7	0.184688
8	0.22367
9	0.213098
10	0.31047

Table S2. *Cont.*

Number of Features	*MCC* Value
11	0.31511
12	0.335302
13	0.337675
14	0.371441
15	0.357833
16	0.30472
17	0.321369
18	0.364566
19	0.30472
20	0.321369
21	0.36077
22	0.38708
23	0.30472
24	0.321369
25	0.387
26	0.37435
27	0.38703
28	0.33567
29	0.30917
30	0.36104
31	0.44837
32	0.47392
33	0.46171
34	0.50671
35	0.48708
36	0.44838
37	0.44317
38	0.43547
39	0.43558
40	0.4457
41	0.42228
42	0.43304
43	0.43066
44	0.43124
45	0.46823
46	0.49286
47	0.47816
48	0.42737
49	0.49288
50	0.46346
51	0.48041
52	0.42797
53	0.44818
54	0.44996

Table S2. *Cont.*

Number of Features	*MCC* Value
55	0.4451
56	0.47657
57	0.511945
58	0.48017
59	0.47001
60	0.49975
61	0.49995
62	0.42495
63	0.49549
64	0.50193
65	0.42271
66	0.41146
67	0.41871
68	0.41099
69	0.41171
70	0.47517
71	0.36287
72	0.36827
73	0.36413
74	0.36322
75	0.38389
76	0.37354
77	0.28017
78	0.37159
79	0.32978
80	0.37005
81	0.35437
82	0.3726
83	0.32273
84	0.39282
85	0.32724
86	0.33419
87	0.33297
88	0.33257
89	0.33661
90	0.33461
91	0.34751
92	0.31669
93	0.35892
94	0.31154
95	0.37755
96	0.29682
97	0.31636
98	0.39595

Table S2. *Cont.*

Number of Features	*MCC* Value
99	0.37356
100	0.36403
101	0.389527
102	0.34074
103	0.3462
104	0.33837
105	0.3417
106	0.35658
107	0.369136
108	0.31853
109	0.374633
110	0.33882
111	0.31004
112	0.343
113	0.39283
114	0.30999
115	0.39414
116	0.37616
117	0.31685
118	0.30156
119	0.32367
120	0.33008
121	0.35094
122	0.34973
123	0.34886
124	0.32026
125	0.35052
126	0.35472
127	0.31311
128	0.33733
129	0.34493
130	0.37529
131	0.351216
132	0.34408
133	0.353342
134	0.31957
135	0.36028
136	0.36788
137	0.37178
138	0.32508
139	0.353527
140	0.377682
141	0.378586
142	0.3791

Table S2. *Cont.*

Number of Features	*MCC* Value
143	0.34641
144	0.34859
145	0.35584
146	0.38327
147	0.31594
148	0.288852
149	0.2032
150	0.22272
151	0.21241
152	0.283288
153	0.24002
154	0.20645
155	0.25961
156	0.25627
157	0.20561
158	0.23553
159	0.29219
160	0.293609
161	0.255118
162	0.23875
163	0.28708
164	0.24575
165	0.22018
166	0.21152
167	0.262693
168	0.27785
169	0.21042
170	0.20071
171	0.23835
172	0.20067
173	0.25654
174	0.2285
175	0.28467
176	0.22183
177	0.2208
178	0.24512
179	0.25107
180	0.22012
181	0.2580797
182	0.24243
183	0.298773
184	0.27508
185	0.284172
186	0.252942

Table S2. *Cont.*

Number of Features	*MCC* Value
187	0.22272
188	0.22025
189	0.26595
190	0.29539
191	0.2261
192	0.22752
193	0.24944
194	0.25577
195	0.2524252
196	0.22759
197	0.282479
198	0.27697
199	0.273907
200	0.261227
201	0.21401
202	0.280397
203	0.2974
204	0.25317
205	0.22907
206	0.23758
207	0.274169
208	0.208
209	0.26483
210	0.268931
211	0.262164
212	0.272302
213	0.20888
214	0.2444
215	0.207
216	0.22759
217	0.22095
218	0.23594
219	0.22093
220	0.22338
221	0.286176
222	0.24036
223	0.22917
224	0.24914
225	0.23212
226	0.2007
227	0.23926
228	0.29365
229	0.22692
230	0.28413

Table S2. *Cont.*

Number of Features	*MCC* Value
231	0.22334
232	0.2823
233	0.283045
234	0.27254
235	0.288749
236	0.24535
237	0.265739
238	0.265988
239	0.20031
240	0.20275
241	0.287216
242	0.29377
243	0.21502
244	0.2974
245	0.22221
246	0.260333
247	0.24984
248	0.288167
249	0.22394
250	0.26179
251	0.275798
252	0.24095
253	0.288715
254	0.20449
255	0.20095
256	0.280367
257	0.22689
258	0.2528582
259	0.2588378
260	0.2123
261	0.21229
262	0.2938
263	0.255203
264	0.21833
265	0.271235
266	0.287884
267	0.251067
268	0.23698
269	0.26501
270	0.261862
271	0.277903
272	0.20884
273	0.2582347
274	0.21886

Table S2. *Cont.*

Number of Features	*MCC* Value
275	0.258449
276	0.271218
277	0.28968
278	0.25217
279	0.22599
280	0.26076
281	0.23779
282	0.27556
283	0.265597
284	0.262177
285	0.21446
286	0.272743
287	0.21126
288	0.23198
289	0.20918
290	0.262994
291	0.23208
292	0.229978
293	0.226448
294	0.237417
295	0.20448
296	0.248738
297	0.254565
298	0.21261
299	0.262839
300	0.25651
301	0.21471
302	0.265938
303	0.24263
304	0.25722
305	0.257194
306	0.2123
307	0.2852
308	0.21985
309	0.22258
310	0.22749
311	0.29119
312	0.21558
313	0.2939
314	0.2117
315	0.213777
316	0.2230403
317	0.223808
318	0.21641

Table S2. *Cont.*

Number of Features	*MCC* Value
319	0.21926
320	0.213604
321	0.25156
322	0.238772
323	0.22781
324	0.201656
325	0.210776
326	0.21626
327	0.21773
328	0.24438
329	0.25587
330	0.21471
331	0.22394
332	0.298819
333	0.23237
334	0.283914
335	0.254243
336	0.24095
337	0.21543
338	0.215443
339	0.23951
340	0.223525
341	0.219393
342	0.22836
343	0.2751
344	0.23979
345	0.23568
346	0.24279
347	0.24489
348	0.20066
349	0.235024
350	0.23698
351	0.23951
352	0.21302
353	0.218926
354	0.27723
355	0.210576
356	0.2133
357	0.2635
358	0.29116
359	0.28165
360	0.23734
361	0.21032
362	0.293315

Table S2. *Cont.*

Number of Features	*MCC* Value
363	0.2089
364	0.21177
365	0.289978
366	0.24008
367	0.22432
368	0.2181
369	0.210958
370	0.21159
371	0.284312
372	0.27204
373	0.20591
374	0.200845
375	0.215345
376	0.21466
377	0.291524
378	0.21119
379	0.21722
380	0.261983
381	0.2133
382	0.273801
383	0.20135
384	0.291374
385	0.20252
386	0.20716
387	0.21621
388	0.26
389	0.22481
390	0.29365
391	0.205
392	0.23743
393	0.286111
394	0.2045
395	0.276749
396	0.278924
397	0.2076
398	0.20618
399	0.2929
400	0.22098
401	0.293646
402	0.20466
403	0.20279
404	0.21302
405	0.21655
406	0.255253

Table S2. *Cont.*

Number of Features	*MCC* Value
407	0.21261
408	0.298635
409	0.21714
410	0.286637
411	0.21621
412	0.22867
413	0.287911
414	0.22534
415	0.20935
416	0.26071
417	0.20066
418	0.21864
419	0.21886
420	0.260258
421	0.2574643
422	0.21103
423	0.277022
424	0.277754
425	0.293849
426	0.271889
427	0.295443
428	0.265737
429	0.284312
430	0.28592
431	0.2039
432	0.28592
433	0.2366
434	0.22875
435	0.261113
436	0.265035
437	0.2644725
438	0.29934
439	0.263699
440	0.21913
441	0.22658
442	0.290255
443	0.22369
444	0.277126
445	0.21177
446	0.288561
447	0.26909
448	0.22505
449	0.26459
450	0.270067

Table S2. *Cont.*

Number of Features	*MCC* Value
451	0.28973
452	0.278772
453	0.23614
454	0.292449
455	0.21602
456	0.23279
457	0.28165
458	0.27345

Table S3. Feature selected by Relative Entropy selection and Incremental feature selection. Among the 57 optimal feature subsets, 48 belonged to the evolutionary conservation scores (shown in red); 3 to the predicted secondary structure (shown in black); 6 to the physicochemical properties (shown in blue).

No.	Feature Index	Description
1	440	Unfolding enthalpy change of chain
2	295	P/P
3	146	G/Q
4	148	G/G
5	416	Molecular weight
6	442	Turn tendency
7	85	C/C
8	64	D/D
9	127	E/E
10	22	R/R
11	274	F/F
12	211	L/L
13	204	L/D
14	206	L/Q
15	208	L/G
16	436	Surrounding hydrophobicity
17	203	L/N
18	379	Y/Y
19	1	A/A
20	169	H/H
21	441	Solvent accessible reduction ratio
22	358	W/G
23	232	K/K
24	43	N/N
25	150	G/I
26	151	G/L
27	413	Beta-helical tendency
28	337	T/T
29	402	$Total\_number_H$
30	125	E/C

Table S3. *Cont.*

No.	Feature Index	Description
31	78	D/W
32	316	S/S
33	400	V/V
34	190	L/I
35	215	L/P
36	209	L/H
37	405	Average_length_H
38	207	L/E
39	298	P/W
40	294	P/F
41	212	L/K
42	216	L/S
43	160	G/V
44	186	I/Q
45	188	I/G
46	386	Y/Q
47	388	Y/G
48	384	Y/D
49	71	D/L
50	134	E/F
51	130	E/I
52	154	G/F
53	18	A/W
54	401	Total_number_C
55	389	V/H
56	159	G/H
57	65	D/C

Table S4. The predicted results by PSNO for Xue's independent dataset. The first column shows the UniProt ID for the protein and the second whether a SNOs is predicted.

UniProt ID	The Site Is Deteced?
Q9UPN3	YES
O88307	YES
P29803	NO
Q9UPN3	YES
P42345	YES
P31040	YES
Q9NYC9	YES
Q9SJU4	YES
Q8IWV7	YES
P54265	YES
O75369	YES
Q96RW7	YES
Q96RW7	YES
P00505	YES

Table S4. *Cont.*

UniProt ID	The Site Is Deteced?
Q8WZ42	YES
P35052	YES
P49454	YES
Q5VST9	YES
O43374	YES
P07310	YES
Q8WZ42	YES
P54652	YES
Q8WZ42	YES
Q5VST9	YES
Q96RW7	YES
Q9P1W8	YES
P06396	YES
P48643	YES
Q96FJ0	YES
Q8WZ42	NO
O88307	YES
P07900	YES
P54609	YES
Q99798	YES
Q8WZ42	YES
P02787	YES
Q9NYC9	YES
Q8WXH0	YES
P10515	YES
P60900	YES
Q8NBX0	YES
P21333	YES
P14625	YES
Q8WZ42	YES
P35499	YES
B4DX73	YES
P02788	YES
Q8WZ42	YES
Q96FJ0	YES
Q8WZ42	YES
Q8WZ42	YES
P78527	YES
P04406	YES
P15924	YES
Q92793	YES
P27140	YES
Q8WZ42	YES
P34791	YES

Table S4. *Cont.*

UniProt ID	The Site Is Deteced?
Q9H3G5	YES
B4DX73	YES
Q8TE82	YES
Q8WZ42	YES
P38117	YES
Q8WZ42	YES
Q8WZ42	YES
Q14524	YES
Q8WZ42	YES
Q8TDR2	YES
Q9UPN3	YES
P27708	YES
Q8WXH0	YES
O14645	YES
O43933	YES
Q9CWS0	YES
P49454	YES
P24539	YES
P19367	YES
Q96RW7	YES
P12883	YES
Q8WZ42	YES
P23458	YES
P62258	YES
Q8WXH0	YES
P54609	YES
Q99798	YES
Q8WZ42	NO
Q8TE82	YES
P43490	YES
Q6PKC3	YES
Q8NBX0	YES
A2AUS0	YES
Q8WZ42	YES
P25705	YES
P49327	YES
Q64521	YES
P31146	YES
Q9NYC9	YES
Q93008	YES
Q6UB99	NO
Q8WZ42	YES
Q6IA69	YES
Q9UPN3	YES
Q6AWV3	YES

Table S4. *Cont.*

UniProt ID	The Site Is Deteced?
Q9UPA5	YES
Q9P1W8	YES
Q96RW7	YES
P10795	YES
O75179	YES
P78527	YES
Q99996	YES
Q8NEZ4	YES
Q8WZ42	YES
Q460N5	YES
P07724	YES
Q8NEB7	YES
P10796	YES
Q8WZ42	YES
Q9SKP6	YES
Q96HH9	YES
Q92793	YES
P21333	YES
Q9S7I3	YES
Q13509	YES
Q9ZP05	YES
Q9Y6V0	YES
Q9WV42	YES
Q16555	YES
Q8WZ42	YES
Q13509	YES
Q9R158	YES
Q5VST9	YES
Q6UB99	NO
Q8WZ42	YES
Q9ZR03	YES
B4DX73	YES
Q9BYZ2	YES
Q15149	YES
P07724	YES
P04637	YES
Q8WXH0	YES
Q8WZ42	YES
P15924	YES
Q8WZ42	YES
Q92793	NO
O75179	YES
O88307	YES
Q5VST9	YES
Q13535	YES

Table S4. *Cont.*

UniProt ID	The Site Is Deteced?
Q9LUT2	YES
P11142	YES
P98064	YES
P12110	YES
Q9Y265	YES
P56777	NO
P02768	YES
Q9UPN3	YES
P07288	YES
Q8WXH0	YES
Q8WXH0	YES
Q12955	YES
P26358	YES
P12883	YES
O60437	YES
Q6PKC3	YES
Q8WZ42	YES
P35052	YES
Q8N427	YES
Q6IA69	YES
Q8IV32	YES
P00747	YES
Q96Q15	NO
Q8WZ42	YES
P09972	YES
P53492	YES
Q43746	YES
Q8WZ42	YES
Q7Z4H7	YES
O75390	YES
Q13535	YES
Q5VST9	YES
O75179	YES
P17066	YES
Q8WXH0	YES
O75179	YES
Q8WZ42	YES
P49327	YES
P35499	YES
Q71U36	YES
Q93008	YES
Q8WZ42	YES
P00747	YES
O75179	YES

Table S4. *Cont.*

UniProt ID	The Site Is Deteced?
O60309	YES
P02787	YES
Q8WZ42	YES
Q8WXH0	YES
O75037	YES
Q9UPN3	YES
Q9SKP6	YES
Q13939	YES
Q8WZ42	YES
Q8IWV7	YES
Q12931	YES
P12883	YES
O75037	YES
P49454	YES
P26358	YES
Q8WXH0	YES
Q9Y2Q0	YES
Q5VST9	YES
Q8WZ42	YES
Q5VST9	YES
Q8WZ42	YES
Q9LJX4	NO
P49454	YES
Q99798	YES
Q92736	YES
P78527	YES
Q460N5	YES
Q9SRV5	YES
P54265	YES
P09622	NO
P12883	YES
O43374	YES
Q8WZ42	YES
Q8IV32	YES
P51818	YES
P78527	YES
Q9NYC9	YES
O88307	YES
P19367	YES
Q9P2Q2	YES
P42345	YES
Q96RW7	YES
Q13576	YES
Q93008	YES

Table S4. *Cont.*

UniProt ID	The Site Is Deteced?
P26358	YES
Q9BYJ4	NO
P37040	YES
O43374	YES
P02768	YES
Q8WWK9	YES
P22954	YES
Q9Y2Q0	YES
Q96Q15	YES
O43933	YES
P07900	YES
Q99K10	YES
Q9Y6L6	YES
P11177	YES
Q8WYR1	NO
P27612	YES
Q8WWI5	YES
Q8WZ42	YES
P54609	YES
Q8NEZ4	NO
Q9ULB1	YES
O95271	YES
Q5JRA6	YES
Q9WV42	YES
Q99LC5	YES
P12883	YES
P60174	YES
P00747	YES
P26232	YES
Q8NEZ4	YES
Q9BS86	YES
Q92793	YES
P94072	YES
O75179	YES
Q9UPN3	YES
Q9Y6Z4	YES
Q9Y230	YES
Q01955	YES
Q8WZ42	YES
Q5JRA6	YES
Q13576	YES
Q9UPA5	YES
P02787	YES
Q13535	YES

Table S4. *Cont.*

UniProt ID	The Site Is Deteced?
P35499	YES
P23458	YES
Q8WZ42	YES
Q9BUF5	YES
Q16658	YES
Q5JRA6	YES
P42345	YES
Q92793	YES
P63101	NO
Q8WZ42	YES
P26641	YES
Q93008	YES
P60709	YES
Q96RW7	NO
Q96RW7	YES
Q8WZ42	YES
Q02952	YES
P10323	YES
Q5JQC9	YES
P62873	YES
P07724	NO
P42357	YES
Q6JEL2	YES
Q460N5	YES
Q9R158	YES
Q8WZ42	YES
Q8WZ42	YES
Q9S7E7	YES
P07900	YES
P12110	YES
O14715	YES
Q8TDR2	YES
Q8TDR2	YES
P19171	YES
O08759	YES
P07947	YES
Q9S7I3	YES
Q16555	YES
O75952	YES
P29803	NO
P78527	YES
P62736	YES
P25856	YES
Q8IWV7	YES

Table S4. *Cont.*

UniProt ID	The Site Is Deteced?
Q93008	YES
Q8WZ42	YES
Q9ULB1	YES
P21796	YES
Q8WWK9	YES
Q5VST9	YES
Q8WWK9	YES
Q96RW7	YES
Q8NDX6	YES
O95861	YES
Q96RW7	YES
Q8WZ42	YES
Q96RW7	YES
Q38946	YES
Q8WZ42	YES
O75179	YES
Q9NQ38	YES
P50990	YES
P98156	YES
Q9SW21	YES
P12532	YES
P02768	YES
Q8WZ42	YES
Q92777	YES
Q5VST9	YES
Q92736	YES
Q9NYC9	YES
Q9ULB1	NO
Q13618	YES
P00568	YES
P00558	YES
Q9H489	YES
O88307	NO
Q8WZ42	YES
Q9ULB1	YES
Q8WZ42	YES
Q9UPA5	YES
P26358	YES
P00558	YES
P21333	YES
P78527	YES
P00747	YES
Q14008	YES
P31146	YES

Table S4. *Cont.*

UniProt ID	The Site Is Deteced?
Q64521	YES
Q460N5	YES
Q9NYC9	YES
P04075	YES
Q9LHA8	YES
Q96RW7	YES
P15924	YES
Q8NEB7	YES
Q13535	YES
P15924	YES
Q9LJX4	YES
Q6UB99	YES
P07724	YES
P60900	YES
Q16698	YES
Q96RW7	YES
P50851	YES
Q9UKU0	YES
P38117	YES
O43719	YES
Q8R081	YES
P10796	YES
Q99666	YES
Q96RW7	YES
P26232	YES
Q0WL92	YES
P50851	YES
P63261	YES
O95271	YES
Q8WXH0	YES
Q9BYZ2	YES
O65396	YES
P29511	YES
Q8WZ42	YES
Q99996	YES
Q9NYC9	YES
P02787	YES
O75369	YES
Q8R081	YES
Q8WZ42	YES
Q9WV42	YES
Q92736	YES
Q9S841	YES
O23255	YES

Table S4. *Cont.*

UniProt ID	The Site Is Deteced?
P60174	YES
Q92922	YES
Q13576	YES
Q5VST9	YES
P00338	YES
Q8WZ42	YES
P78527	NO
P31040	YES
Q9D8L4	YES
P02787	YES
Q8WWI5	NO
P54609	YES
Q5VST9	YES
Q96RW7	YES
Q8WZ42	YES
P49454	YES
O95271	YES
Q99250	YES
Q9P2Q2	YES
Q38946	NO
P78527	YES
Q8WZ42	YES
P07724	YES
Q96RW7	YES
Q92736	YES
Q8WZ42	YES
Q15149	NO
Q6UB99	YES
P38117	YES
Q9Y6V0	YES
P50851	YES
P49327	YES
Q9H4A3	YES
Q9SRV5	YES
Q96Q15	NO
Q9ASR0	YES
Q9BYJ4	YES
Q6JEL2	YES
P35499	YES
P09622	YES
P26358	YES
Q9H4B7	YES
Q8WZ42	YES
Q8WZ42	YES

Table S4. *Cont.*

UniProt ID	The Site Is Deteced?
P10896	YES
Q92526	YES
Q15149	YES
Q8WWI5	YES
P18669	YES
Q8WZ42	YES
Q99K10	YES
P50851	YES
P27708	YES
Q9SUR0	YES
Q8WZ42	YES
O95271	YES
Q8IWV7	YES
Q6UB99	YES
Q13283	YES
Q99250	NO
P13639	YES
P49454	YES
P27323	YES
P12532	YES
Q8WZ42	YES
Q01955	NO
Q8WZ42	YES
Q13535	NO
Q8WZ42	YES
P42813	YES
Q5JQC9	YES
P07288	YES
P45952	YES
Q13535	YES
Q14315	YES
P11142	YES
P17987	YES
Q8WZ42	YES
Q12955	YES
Q96FJ0	YES
O15027	YES
Q92736	YES
Q9UPN3	YES
Q8VY03	YES
Q8WZ42	YES
O88307	YES
P10323	YES
P53492	YES

Table S4. *Cont.*

UniProt ID	The Site Is Deteced?
P61163	YES
P35235	YES
Q9Y6L6	YES
P27323	YES
Q96RW7	YES
P27612	YES
Q93008	YES
Q99996	YES
Q9EQN8	YES
Q8WXH0	YES
P07205	YES
Q8NEZ4	YES
Q96RW7	YES
O95886	YES
O60437	YES
Q12931	YES
P14625	YES
P49454	YES
P00747	YES
P78527	YES
P23458	YES
Q5VST9	YES
Q12955	YES
P55072	YES
Q8WZ42	YES
Q8W4H7	YES
P02768	YES
A8MRZ7	YES
Q6JEL2	YES
O75369	YES
P02787	YES
Q9ULB1	YES
Q13576	YES
Q92793	YES
Q9UPN3	YES
Q8WXH0	YES
Q8R081	YES
Q12955	YES
Q8VY03	YES
Q6UVJ0	YES
P78559	YES
P48491	YES
P49327	YES
P00747	NO

Table S4. *Cont.*

UniProt ID	The Site Is Deteced?
O88307	YES
Q9SXJ7	YES
Q96RW7	YES
Q8WZ42	YES
Q5TZA2	YES
Q9UPN3	YES
Q9SGT4	YES
O23255	YES
Q8WZ42	YES
Q96RW7	YES
Q96RW7	YES
P21333	YES
Q93008	YES
P02787	YES
P08238	YES
P30101	YES
B1AVT9	YES
Q9UPN3	YES
Q9LPW0	YES
Q96Q15	YES
P27612	YES
P02788	YES
O75179	YES
Q8WZ42	YES
Q540M5	YES
P10797	YES
Q12931	YES
P40939	YES
Q13618	YES
O88307	YES
Q8NEZ4	NO
Q8WZ42	YES
Q12955	YES
Q9Z0L3	NO
Q9D8L4	YES
P27708	YES
Q01955	NO
Q8NEB7	YES
P02788	YES
Q9SGT4	YES
Q99250	YES
Q93008	YES
Q99996	YES

Table S4. *Cont.*

UniProt ID	The Site Is Deteced?
Q9ZRW8	YES
P09972	YES
Q8WZ42	YES
Q9R158	NO
Q13509	YES
Q8NEZ4	YES
P26358	YES
P36873	YES
P49454	YES
Q8R4I4	YES
Q8WZ42	YES
P30042	YES
P61981	YES
Q9BYJ4	YES
P42813	YES
Q99250	YES
Q14524	YES
P15924	YES
Q92820	YES
O43374	YES
Q8WZ42	YES
Q8NEZ4	YES
Q86YZ3	YES
Q9UPN3	YES
Q14566	NO
Q96RW7	YES
P78527	YES
Q99798	YES
Q94BT9	YES
P78527	YES
Q12955	YES
P42345	YES
Q9H4A3	YES
P14618	YES
Q6P8J7	YES
Q8WZ42	YES
P98156	YES
Q8WZ42	YES
P98156	NO
Q92526	YES
Q8WZ42	YES
Q13535	YES
Q9UPN3	YES
Q8WZ42	YES

Table S4. *Cont.*

UniProt ID	The Site Is Deteced?
Q8IWV7	YES
P62191	YES
Q8WZ42	YES
Q93008	YES
Q5VST9	YES
O88307	NO
Q99K10	YES
P07205	YES
Q8WZ42	YES
Q92736	YES
Q8WZ42	YES
P00747	YES
P29803	YES
Q6Z8D9	YES
B1AVT9	NO
P42345	YES
O75369	YES
O88307	YES
Q8WZ42	YES
Q8WZ42	YES
P27323	YES
Q96RW7	YES
P35499	YES
Q12955	YES
P29197	YES
B9DGD1	YES
Q9UPN3	YES
Q93008	YES
Q8VXW1	YES
P07900	YES
Q9ZP05	YES
Q96RW7	YES
Q12955	YES
P53814	YES
Q8WZ42	YES
P10323	YES
P12532	YES
P26358	YES
P31948	YES
P49189	YES
Q6P8J7	YES
Q13748	YES
Q8WZ42	YES

Table S4. *Cont.*

UniProt ID	The Site Is Deteced?
Q9ZSK4	YES
Q13618	YES
O14715	YES
P93819	YES
Q8WZ42	YES
Q8TDY3	YES
P02788	YES
Q6JEL2	YES
Q8WZ42	YES
P26358	YES
Q8WXH0	YES
Q9UPN3	YES
Q8WWI5	YES
Q9UPN3	YES
Q9UPN3	YES
Q8WXH0	YES
P98064	YES
Q9NYC9	YES
Q96HH9	YES
Q9NYC9	YES
Q14315	YES
Q9UPN3	YES
Q5VST9	YES
Q5VST9	YES
Q9SU69	YES
Q8WZ42	YES
P15104	YES
P54609	YES
Q92945	YES
P13639	YES
P38646	YES
P49327	YES
P21333	YES
P54652	YES
P78559	YES
Q9ZP06	YES
P17987	YES
P07724	YES
P07195	NO
P27708	YES
O75179	YES
P68371	YES
P23458	YES
Q8WZ42	YES

Table S4. *Cont.*

UniProt ID	The Site Is Deteced?
P31946	YES
Q8WZ42	YES
Q8NEZ4	YES
Q99250	YES
P48491	YES
P49327	YES
P45952	NO
Q8S9L5	YES
Q13748	YES
P07310	YES
Q61344	YES
P78527	YES
Q9LZY8	YES
Q9SKP6	YES
Q01955	YES
Q6IA69	NO
Q8WZ42	YES
P78559	YES
Q3TJ94	YES
Q8IWV7	YES
Q14524	YES
P62736	YES
Q8WWI5	NO
A2AUS0	YES
Q8WWK9	YES
P17661	YES
Q92793	NO
Q99250	YES
P50851	YES
Q9SYT0	YES
Q9UPA5	YES
P50851	YES
P98156	YES
P13639	YES
Q9ULW0	YES
Q9R158	YES
P19367	YES
Q3TJ94	YES
Q56ZK3	YES
P62873	YES
Q8WZ42	YES
P07724	YES
Q9ULW0	YES
P23368	YES

Table S4. *Cont.*

UniProt ID	The Site Is Deteced?
P25696	YES
Q14524	YES
Q9Y6V0	YES
Q96RW7	YES
Q8WZ42	YES
Q8H0B7	YES
P28066	YES
P21333	YES
Q8NEZ4	YES
P23458	YES
Q8WZ42	YES
Q8WZ42	YES
Q8R081	YES
P31146	YES
O43374	YES
Q14990	YES
Q9UPN3	YES
P42813	YES
P61106	YES
P15924	YES
Q8WZ42	YES
Q8WYR1	NO
Q14315	YES
P26358	YES
Q13535	YES
Q99798	YES
P27708	YES
Q14566	YES
Q9S7E7	YES
Q9FNE2	YES
Q5VST9	YES
P50851	NO
Q8WZ42	YES
O88307	YES
Q8WZ42	YES
P17987	YES
P63104	NO
Q949U7	YES
Q8NEB7	YES
Q9UJ83	YES
Q96RW7	YES
Q9UI46	YES
Q13535	YES
Q8WZ42	YES

Table S4. *Cont.*

UniProt ID	The Site Is Deteced?
Q9Z0L3	YES
Q92793	YES
Q99M47	YES
P39207	YES
O75369	YES
Q9Y277	YES
Q9LKA3	YES
Q8WZ42	YES
P14618	YES
P78527	YES
O95861	YES
P98064	YES
Q460N5	YES
Q8WZ42	YES
Q8WZ42	YES
P27708	YES
O88307	YES
O75037	YES
Q8WXH0	YES
P49454	YES
Q03265	YES
Q9SXJ7	YES
Q9D8L4	YES
P25697	YES
P00505	NO
P31948	YES
P78527	YES
Q6IA69	YES
Q96RW7	YES
Q8TE82	NO
O15027	YES
P17987	YES
Q9H4A3	YES
P04637	YES
Q93008	YES
P43155	YES
Q9UPN3	YES
O75952	YES
P40939	YES
Q8N1C8	YES
Q8WXH0	YES
Q14566	YES
P45880	YES
P07724	YES

Table S4. *Cont.*

UniProt ID	The Site Is Deteced?
P12270	YES
Q8WXH0	YES
P07724	YES
Q8WZ42	YES
Q9Y2Q0	YES
P56597	YES
Q86UR5	YES
Q8VXH1	YES
Q8WZ42	YES
Q9ULB1	YES
Q6UVJ0	YES
Q9FPF0	YES
Q9EQV1	YES
P11169	YES
Q13011	YES
Q6GU14	YES
Q92526	YES
O88307	YES
Q96FJ0	YES
Q9NYC9	YES
B1AXT2	YES
Q14524	YES
P68363	YES
Q9Y6Z4	YES
Q96RW7	YES
O88307	NO
Q96FJ0	YES
Q14008	YES
Q12955	YES
P02788	NO
P04264	YES
P42345	YES
P21333	YES
Q9UJ83	YES
Q6UB99	YES
O14715	YES
Q91VD9	YES
Q8WZ42	YES
Q8WZ42	YES
Q8WZ42	YES
Q92945	YES
Q9H8N7	YES
O43933	YES
Q91VD9	YES

Table S4. *Cont.*

UniProt ID	The Site Is Deteced?
Q8IWV7	YES
Q9SU69	YES
Q9UPN3	YES
P50851	YES
Q8WWI5	YES
Q593N9	YES
P15313	YES
Q8WZ42	YES
Q8NEZ4	NO
P48643	YES
Q8WZ42	YES
Q8WZ42	YES
Q13748	YES
P50990	YES
Q8WXH0	YES
P30101	YES
Q99M47	YES
P07237	YES
O14645	YES
O75369	YES
Q96RW7	YES
P17987	YES
O43719	YES
P00558	YES
P68371	YES
Q8NEZ4	YES
O88307	YES
P78527	YES
Q8H0B7	YES
A2AUS0	YES
O88307	NO
Q12955	YES
P49327	YES
Q8WZ42	YES
Q9LR30	YES
Q8WZ42	YES
Q6IA69	YES
Q9LF98	YES
P38646	YES
Q8WZ42	YES
Q6UB99	YES
B1AXT2	YES
Q92922	YES
Q15149	YES

Table S4. *Cont.*

UniProt ID	The Site Is Deteced?
P02768	YES
Q99250	YES
Q01955	YES
P31146	YES
Q96RW7	YES
Q12955	YES
Q14315	YES
Q8WZ42	YES
P15924	YES
Q92736	YES
P42345	YES
Q16555	YES
P78527	YES
P21266	YES
Q5VST9	YES
Q3TRK3	YES
P78527	YES
Q9UKU0	YES
Q8NDH3	YES
O14556	YES
P07724	YES
Q8NEZ4	YES
P17174	YES
Q92793	YES
Q8R4I4	YES
Q5JRA6	YES
P49454	YES
P49454	YES
P10323	YES
P26358	YES
Q5VST9	YES
Q8WZ42	YES
Q8WZ42	YES
B1AVT9	YES
P25696	YES
O49485	YES
Q86UR5	YES
Q8WXH0	YES
P15104	YES
Q8TE82	YES
Q99M47	YES
Q13535	YES
Q8WZ42	YES
Q5VST9	YES

Table S4. *Cont.*

UniProt ID	The Site Is Deteced?
P02787	YES
P28066	YES
Q9LPW0	YES
Q8WZ42	YES
Q9SU69	YES
Q9UPN3	YES
Q96RW7	YES
P47998	YES
Q9Y277	YES
O95271	YES
Q5JQC9	NO
P49454	YES
Q6GU14	YES
Q92526	YES
Q96ME7	YES
P15104	YES
P31937	YES
Q15149	YES
Q16658	YES
Q9S7E7	YES
Q8WZ42	YES
B1AXT2	YES
Q15149	YES
Q8WZ42	YES
Q9NYC9	YES
P49454	YES
Q460N5	YES
P49454	YES
Q8WXH0	YES
Q13509	YES
P09622	YES
Q8WZ42	YES
Q8WZ42	YES
Q9Y2Q0	YES
Q8WWK9	YES
P55072	YES
P55072	YES
Q6UB99	YES
O88307	NO
Q8IWV7	NO
O95861	YES
Q9LR30	YES
O75179	YES
Q8WZ42	YES

Table S4. *Cont.*

UniProt ID	The Site Is Deteced?
P07724	YES
P00918	YES
Q9ULW0	YES
Q6P8J7	YES
P78527	YES
Q8WZ42	YES
Q01813	YES
Q9Y6V0	YES
Q92526	YES
Q5VST9	YES
Q8NEZ4	YES
Q8WXH0	YES
Q91VD9	YES
Q96RW7	YES
Q99250	NO
P27708	YES
P00367	YES
P62873	YES
Q9H8N7	YES
Q93008	NO
Q8N1C8	YES
Q02952	YES
Q9BXM0	YES
P35235	YES
Q9BYJ4	YES
Q92793	YES
P63101	YES
P49454	YES
Q9H3G5	YES
Q8WZ42	YES
Q8S9L5	YES
P23458	YES
Q6IA69	YES
Q9Y265	YES
Q96QE4	YES
P07947	YES
Q93008	YES
O88307	NO
P68033	YES
P08752	YES
P56777	YES
Q9Y6V0	YES
A2AUS0	YES
Q9SU69	YES

Table S4. *Cont.*

UniProt ID	The Site Is Deteced?
Q8WZ42	YES
Q06830	YES
Q9NYC9	YES
Q9P1W8	YES
P27708	YES
O88307	YES
P35499	NO
P47998	YES
Q99250	YES
Q8WWK9	YES
Q13885	YES
P49327	YES
Q8WXH0	YES
Q9UPN3	YES
P21266	YES
Q96RW7	YES
Q5VST9	YES
Q8WZ42	YES
Q8R081	YES
P98156	NO
Q13939	YES
P48047	YES
P29197	YES
Q8NDX6	YES
P23458	NO
Q8WZ42	YES
P10896	YES
Q9S841	YES
Q9SIB9	YES
O48646	YES
O08759	YES
O88307	YES
Q16658	YES
Q9R158	NO
P35052	YES
P40939	YES
Q8WZ42	YES
Q9UJ83	YES
A2AUS0	YES
Q14008	YES
Q99LC5	YES
Q8WZ42	YES
P78527	YES
P49454	YES

Table S4. *Cont.*

UniProt ID	The Site Is Deteced?
Q8WZ42	YES
Q8WZ42	YES
Q6Z8D9	YES
Q6UB99	YES
Q5VST9	YES
P19367	YES
Q7Z4H7	YES
Q9SXJ7	YES
Q12955	YES
Q13885	YES
Q8WZ42	YES
P42357	YES
Q0WUV6	YES
Q9H7X3	YES
Q9NYC9	YES
Q7Z4H7	YES
Q12955	YES
O43719	YES
Q13625	YES
Q9NYC9	YES
Q8WZ42	YES
Q92793	YES
Q6UB99	YES
Q9EQV1	YES
Q9SYT0	YES
Q13939	YES
P31946	YES
Q14315	YES
P31948	YES
P62191	YES
Q8WZ42	YES
Q8WXH0	YES
Q9UI46	NO
O75369	YES
P00747	YES
Q9ASR0	YES
Q8WXH0	YES
P15924	YES
Q6JEL2	YES
O43719	YES
Q13535	YES
Q02952	YES
Q8WZ42	YES
Q6UVJ0	YES

Table S4. *Cont.*

UniProt ID	The Site Is Deteced?
Q8WZ42	YES
P13639	YES
P35499	NO
Q6JEL2	YES
P78559	YES
P30101	YES
Q96RW7	YES
Q8IWV7	YES
Q9NVA2	YES
Q9LJE4	YES
Q96Q15	YES
Q8WZ42	YES
Q9SIB9	YES
Q5VST9	YES
Q9NYC9	NO
Q5VST9	YES
Q12955	YES
Q9Y5R2	YES
P30042	YES
P00338	NO
Q6JEL2	YES
Q8WZ42	YES
Q5VST9	YES
P62258	YES
Q9UI46	YES
P60709	YES
Q8TE82	YES
Q92793	YES
Q949U7	YES
Q8NEZ4	NO
P68371	YES
Q2HIV2	YES
Q7Z4H7	YES
Q9UJ83	YES
Q9BS86	YES
P81605	YES
Q8TAA3	YES
P00367	YES
O14645	YES
P68033	YES
Q92793	YES
O15027	YES
P68033	YES
Q5JQC9	YES

Table S4. *Cont.*

UniProt ID	The Site Is Deteced?
Q8WZ42	YES
Q8WZ42	YES
Q9UPN3	YES
P02787	YES
P54265	YES
Q9NYC9	YES
Q8WZ42	YES
P36873	YES
Q92736	YES
O75369	YES
Q92793	YES
Q9NYC9	NO
P02788	YES
P23458	YES
O75037	YES
Q42029	YES
Q14315	YES
Q9Y6L6	YES
P02788	YES
Q99LC5	YES
O14715	YES
Q12955	YES
Q6JEL2	YES
P23458	YES
P25696	YES
P21333	YES
P50990	YES
P61163	YES
P31937	YES
P19171	YES
Q5VST9	YES
Q5VST9	YES
Q9BS86	YES
Q8WZ42	YES
O60309	YES
Q9Y6V0	YES
Q14008	YES
Q8WZ42	YES
O43933	YES
P15924	YES
Q96RW7	YES
P78527	YES
P78527	YES
P15924	YES

Table S4. *Cont.*

UniProt ID	The Site Is Deteced?
P27708	YES
Q5VST9	YES
P15924	YES
Q92736	YES
Q3LXA3	YES
P16152	YES
P68363	YES
Q8WZ42	YES
Q8WZ42	YES
P98064	YES
Q93008	YES
Q91VD9	YES
Q9UKU0	YES
P15104	YES
Q5TZA2	YES
O23255	YES
Q99250	YES
Q9H7X3	YES
P49454	YES
Q01955	YES
P30042	YES
Q7Z4H7	YES
O75369	YES
P24704	YES
P12270	YES
O49485	YES
Q6PKC3	NO
Q0WL92	YES
P98156	YES
P62873	YES
Q7Z4H7	YES
P24752	YES
P00747	YES
Q8WXH0	YES
Q14524	YES
Q8WZ42	YES
Q9BUF5	YES
Q8NEZ4	YES
P02768	YES
Q9Y6L6	YES
Q9UI46	YES
Q99996	YES
Q460N5	YES
Q96RW7	YES

Table S4. *Cont.*

UniProt ID	The Site Is Deteced?
Q8WZ42	YES
Q9Y6Z4	YES
Q9UPN3	YES
Q9UPA5	YES
P93819	YES
Q92736	YES
P98156	YES
A8MRZ7	YES
P09622	YES
Q14315	YES
P09542	YES
Q9ULW0	YES
Q8WZ42	YES
P50851	YES
O88307	YES
Q96RW7	YES
Q8WZ42	YES
Q8WZ42	YES
P06748	YES
P13861	YES
O88307	NO
Q5VST9	YES
P13639	YES
P27612	YES
Q86UR5	YES
P51818	YES
P50851	YES
Q5VST9	YES
O88307	NO
Q460N5	YES
Q14315	YES
P17745	YES
O88307	YES
Q9Y6V0	YES
Q14566	YES
Q8WZ42	YES
P25787	YES
Q8WZ42	YES
Q8NBX0	YES
Q8WZ42	YES
P06396	YES
Q8WZ42	YES
Q92736	YES
Q8WZ42	YES

Table S4. *Cont.*

UniProt ID	The Site Is Deteced?
P50851	YES
Q99250	YES
Q8WZ42	YES
Q9NYC9	YES
P23458	YES
P05091	YES
Q8WZ75	YES
P50851	YES
Q14008	YES
Q8WXH0	YES
P02787	YES
Q9H3G5	YES
P14618	YES
Q8VY03	YES
Q8WZ42	YES
Q9SGT4	YES
B4DX73	YES
O43719	YES
Q460N5	YES
Q92820	YES
Q96RW7	YES
Q9ZP06	YES
P07310	YES
Q8WZ42	YES
P00747	YES
Q9SU69	YES
Q8WZ42	YES
Q8WXH0	YES
P13861	YES
Q8WZ42	YES
Q14566	YES
P02787	YES
Q9SA52	YES
Q6JEL2	YES
P25856	YES
Q9H4B7	YES
P02768	YES
Q99K10	YES
Q9ZR03	YES
P07864	YES
Q6UB99	YES
Q8NEZ4	YES
Q13535	YES
P63104	YES

Table S4. *Cont.*

UniProt ID	The Site Is Deteced?
Q92736	NO
P23368	YES
P26358	YES
Q13939	YES
Q3TJ94	YES
Q93008	YES
Q9ULB1	YES
Q9EQV1	YES
P35499	YES
P31040	YES
Q92781	YES
Q0WL92	YES
P50851	YES
Q9NQ38	YES
Q96Q15	YES
P17612	YES
Q96Q15	NO
Q8WZ42	NO
Q86UR5	YES
Q14008	YES
Q5VST9	YES
Q9R158	YES
P23458	YES
P78527	YES
O75179	NO
P00505	YES
Q92793	YES
Q96RW7	YES
P06396	YES
Q8WZ42	YES
Q9Y6V0	YES
P15924	YES
Q92793	YES
P15924	YES
Q99447	YES
Q9Y5R2	YES
Q9FNE2	YES
P49454	YES
Q38946	YES
Q9NYC9	YES
Q02383	YES
P49454	YES
P31948	YES
Q8WZ42	YES

Table S4. *Cont.*

UniProt ID	The Site Is Deteced?
O75969	YES
Q9P1W8	YES
P00747	YES
O95271	YES
Q12955	YES
Q9UKU0	YES
P15104	YES
P34931	YES
P35052	YES
P26232	YES
Q8WZ42	YES
P49189	YES
Q96RW7	YES
Q92736	YES
P40925	YES
Q8WZ42	YES
Q99250	YES
Q8WZ42	YES
Q8WZ42	YES
P00747	YES
Q8WZ42	YES
P06733	YES
Q9UPN3	YES
P12883	YES
Q96Q15	YES
Q9UPA5	YES
Q94BS2	YES
Q9UPN3	YES
Q15149	YES
Q8WZ42	YES
P17987	YES
P15924	YES
O75969	YES
Q8WZ42	YES
O75037	YES
Q8WZ42	YES
Q8NEZ4	YES
Q8WWI5	YES
Q8WZ42	YES
Q3TJ94	YES
Q9ULW0	YES
P98156	YES
P07954	YES
P54609	YES

Table S4. *Cont.*

UniProt ID	The Site Is Deteced?
P37040	YES
P07947	NO
Q9SA56	YES
O88307	YES
P54609	YES
Q9ASR0	YES
Q96RW7	YES
P98156	NO
P78559	YES
P43490	YES
Q8NEB7	YES
P50851	YES
Q8TE82	YES
Q71U36	YES
Q5JRA6	YES
Q8WZ42	YES
P00505	YES
Q9H4B7	YES
Q5VST9	YES
O75390	YES
Q9Y230	YES
Q5VST9	YES
Q460N5	YES
P02787	YES
P55072	YES
Q8R081	YES
Q9UPN3	YES
Q99996	YES
Q5SS40	YES
Q9SGT4	YES
P10797	YES
P06744	NO
Q92736	YES
Q9ZR03	YES
P49454	YES
P54609	YES
Q96RW7	YES
Q15424	YES
P14136	YES
Q8WZ42	YES
Q13011	YES
Q5VST9	YES
Q8WZ42	YES
Q9SA52	YES

Table S4. *Cont.*

UniProt ID	The Site Is Deteced?
P00747	YES
Q9M7T0	YES
Q13535	YES
O75969	YES
Q5VST9	YES
Q9SU69	YES
P19366	YES
P50851	YES
P51818	YES
P68033	YES
P50883	YES
O14715	YES
Q14315	YES
Q14990	YES
Q8WXH0	YES
Q8IWV7	YES
P48036	YES
P15924	YES
Q12955	YES
Q14524	YES
Q460N5	YES
P49327	YES
Q96Q15	NO
Q9Y230	YES
Q8WZ42	YES
P12270	YES
Q9ULB1	YES
Q14524	YES
Q8WZ42	YES
O75037	NO
Q8WWI5	NO
Q01955	YES
O94769	YES
P00747	YES
P28838	YES
Q14008	YES
Q8WYR1	YES
Q9NYC9	YES
Q9BYJ4	YES
O43719	YES
Q92736	YES
Q01955	NO
O82660	YES
Q9NYC9	YES

Table S4. *Cont.*

UniProt ID	The Site Is Deteced?
P98156	YES
Q14008	YES
Q92793	YES
Q9R158	NO
Q5VST9	YES
Q8WZ42	YES
P78527	YES
Q5VST9	YES
Q9NYC9	YES
Q14315	YES
Q9P2Q2	YES
P98156	NO
Q3TJ94	YES
P45880	YES
Q8WZ42	YES
Q8WZ42	YES
Q9SKP6	YES
Q9SIB9	YES
Q9NYC9	YES
Q6ZMR3	YES
Q8WZ42	YES
P51818	YES
Q9BYZ2	YES
Q92526	YES
Q8WXH0	YES
P48643	YES
Q91VD9	YES
P36873	YES
Q8TE82	YES
Q9R158	YES
P21333	YES
P50851	YES
P23458	YES
Q8WZ42	YES
Q6ZMR3	YES
Q13136	YES
Q9UPN3	YES
Q9Z0L3	YES
Q13939	YES
Q8NEZ4	NO
P31040	NO
Q9NQ38	YES
P11177	YES
Q15149	YES

Table S4. *Cont.*

UniProt ID	The Site Is Deteced?
Q8WXH0	YES
Q5VST9	YES
Q9UBX3	YES
Q8WZ42	YES
P02768	YES
Q9Y277	YES
Q8IWV7	YES
Q9BS86	YES
Q9NYC9	YES
P36873	YES
Q86YZ3	YES
Q92793	YES
Q8WZ75	YES
O60309	YES
Q6IA69	YES
Q9ASR0	YES
O14715	YES
Q8WZ42	YES
Q9H7X3	YES
O88307	YES
Q9WV42	YES
Q96Q15	YES
Q01813	YES
Q8W4H7	YES
P26641	YES
Q9NQ38	YES
Q9SU69	YES
Q96RW7	YES
Q6UB99	YES
P15924	YES
Q6PKC3	YES
P10795	YES
Q9H4A3	YES
Q99798	YES
Q8TDR2	YES
P42345	YES
Q9UKU0	YES
Q8IWV7	YES
P49189	YES
Q8WYR1	YES
Q16658	YES
Q91VD9	YES
Q8WXH0	YES
P25788	YES

Table S4. *Cont.*

UniProt ID	The Site Is Deteced?
B1AVT9	YES
P26232	YES
Q5VST9	YES
Q92781	YES
P50990	YES
P07724	YES
O43374	YES
Q61344	YES
Q71U36	YES
Q9UPN3	YES
Q9UPN3	YES
A2AUS0	YES
Q96ME7	YES
Q96RW7	YES
Q15149	YES
P00558	YES
P11021	YES
O23255	YES
Q0WR60	YES
P34791	YES
P15924	YES
P42345	YES
P07237	YES
O08759	NO
Q8WZ42	YES
Q99250	YES
P50851	YES
Q12955	YES
Q8WZ42	YES
P30101	YES
Q5VST9	YES
P31040	YES
O60437	YES
Q8WZ42	YES
Q8WXH0	YES
O43933	YES
P49189	YES
Q9SF85	YES
O75179	YES
P55072	YES
P11169	NO
Q92736	YES
P02787	YES
Q8WZ42	YES

Table S4. *Cont.*

UniProt ID	The Site Is Deteced?
Q8WZ42	YES
P07864	YES
Q9NS25	YES
Q99LC5	YES
Q543S2	YES
Q8NDH3	YES
P10795	YES
P68371	YES
Q9UPA5	YES
Q92736	YES
Q13625	YES
Q14008	YES
P62736	YES
Q41088	YES
P27708	YES
Q8WZ42	YES
P15924	YES
Q8WZ42	YES
Q9ULW0	YES
Q5VST9	YES
Q96LI6	YES
Q8WZ75	YES
Q8NEB7	YES
P27612	YES
Q9UPN3	YES
Q9FPF0	YES
P07724	YES
P34931	YES
P49454	YES
Q96RW7	YES
Q14524	YES
P00747	YES
Q13625	YES
Q8WWI5	YES
P78527	YES
P07724	YES
Q14008	YES
Q99250	YES
P00747	YES
Q92736	YES
P23458	YES
O43374	YES
Q96Q15	YES
Q460N5	YES

Table S4. *Cont.*

UniProt ID	The Site Is Deteced?
P14618	YES
Q8WZ42	YES
O88307	YES
Q92736	YES
P78559	YES
Q9UQ13	YES
P23458	YES
Q8WZ42	YES
Q92736	YES
Q9BUF5	YES
Q14315	YES
Q92526	YES
P19367	YES
Q9FVT2	YES
Q99666	YES
Q8NEZ4	YES
P12110	YES
Q9LJE4	YES
Q9NQ38	YES
Q8IWV7	YES
P35499	YES
P11142	YES
B1AXT2	YES
Q96RW7	YES
Q9ULB1	YES
Q9WV42	YES
P02768	YES
Q8WZ42	YES
Q547G3	YES
Q96FJ0	YES
P02788	YES
Q96RW7	YES
Q13011	YES
O75369	YES
Q96Q15	YES
Q13136	YES
Q9BUF5	YES
O75179	YES
Q9H3G5	YES
Q13535	YES
O65396	YES
Q16658	YES
Q96RW7	YES
P07205	YES

Table S4. *Cont.*

UniProt ID	The Site Is Deteced?
P02768	YES
P26358	YES
P29511	YES
Q9NYC9	YES
P21333	YES
Q5VST9	YES
P00747	YES
Q86UR5	YES
P53814	YES
Q14990	YES
Q99447	YES
Q8WZ42	YES
Q8TAA3	YES
P28161	YES
P49454	YES
P98064	YES
Q93008	YES
Q8WZ42	YES
Q99250	YES
Q9SUR0	YES
Q9NYC9	YES
Q8WZ42	YES
P49454	YES
Q96RW7	YES
Q8R4I4	YES
Q92781	YES
Q7Z4H7	YES
Q8WYR1	YES
Q9FZ06	YES
Q8N1C8	YES
P02787	YES
P15259	YES
Q14008	YES
Q56ZK3	YES
Q8TDY3	YES
Q96RW7	YES
O15027	YES
B4DX73	YES
P49454	YES
Q8WZ42	YES
Q8WXH0	YES
Q8NEZ4	YES
A8MRZ7	YES
Q99996	YES

Table S4. *Cont.*

UniProt ID	The Site Is Deteced?
Q96RW7	YES
Q9SGT4	YES
P07724	YES
Q13618	YES
Q99996	YES
P35235	YES
Q8WZ42	YES
Q02952	YES
Q8WZ42	YES
O43374	YES
P48491	YES
O88307	NO
Q15149	YES
P31040	YES
Q5VST9	YES
Q8WZ42	YES
Q6PKC3	YES
O43933	YES
Q9UPN3	YES
Q6ZMR3	YES
P43155	YES
Q93008	YES
O50008	YES
P05091	YES
Q9UJ83	YES
B9DGD1	YES
Q9H8N7	YES
Q96RW7	YES
P11169	YES
P26232	YES
O48646	YES
P08238	YES
Q8TDY3	YES
Q86UR5	YES
Q8IWV7	YES
P42345	YES
Q8WWI5	YES
Q9Y6V0	YES
Q8WZ42	NO
P07947	YES
P98156	YES
P13639	YES
P15104	YES
Q8WZ42	YES

Table S4. *Cont.*

UniProt ID	The Site Is Deteced?
Q3LXA3	YES
Q96RW7	YES
Q8WZ42	YES
P07237	YES
P78527	YES
Q99798	NO
P78527	YES
Q5VST9	NO
Q14524	YES
P78527	YES
P21333	YES
Q5TZA2	YES
Q8WXH0	YES
Q9UKU0	YES
Q9NQ38	YES
Q14315	YES
Q8H0B7	YES
Q8WXH0	YES
Q8WZ42	YES
Q9SU69	YES
Q92736	YES
Q5JQC9	YES
P26641	YES
Q92793	NO
P13861	YES
Q9ZR03	YES
Q9XEX2	YES
Q96QE4	YES
Q9H4A3	YES
O08759	YES
P38646	YES
Q99996	YES
Q7Z4H7	YES
Q9FVT2	YES
Q5VST9	YES
Q8WZ42	YES
P29511	YES
Q9M7T0	YES
P48347	YES
Q9ULB1	YES
Q8WXH0	YES
Q92793	YES
Q92526	YES

Table S4. *Cont.*

UniProt ID	The Site Is Deteced?
Q9BYJ4	YES
P02768	YES
Q8WZ42	YES
Q8WZ42	YES
O95271	YES
Q8WZ42	YES
Q9LD57	YES
P78559	YES
Q5SS40	YES
P09972	YES
P49327	YES
P62873	YES
P63261	YES
Q12955	YES
Q8WZ75	YES
Q3TRK3	YES
Q9Y5R2	YES
P11021	YES
P78527	YES
Q9NYC9	YES
Q14315	YES
O60309	YES
O03042	YES
Q91X86	YES
P16152	YES
A2AUS0	YES
O88307	YES
O50008	YES
Q96Q15	YES
Q8WZ42	YES
Q9NYC9	YES
P45952	YES
P10515	YES
Q14990	YES
P08238	YES
P26358	YES
P55072	YES
Q96QE4	YES
Q547G3	YES
Q8NDX6	NO
Q9SIB9	YES
P21333	YES
Q8WZ42	YES
Q9FZ06	YES

Table S4. *Cont.*

UniProt ID	The Site Is Deteced?
Q9P2E9	YES
Q92736	YES
Q14990	YES
P35235	YES
P08107	YES
P12110	YES
Q99996	YES
P49454	YES
Q8R4I4	YES
Q8NEZ4	YES
P31040	YES
P78559	YES
P27323	YES
Q3TJ94	YES
Q92777	YES
Q13939	YES
P35499	YES
P42345	YES
Q9SU69	YES
Q6JEL2	NO
P27708	YES
Q6GU14	YES
Q9NYC9	NO
Q93008	YES
Q8TE82	YES
Q9LZY8	YES
P78527	YES
Q86UR5	NO
Q5VST9	YES
Q540M5	YES
Q9UPN3	YES
Q13576	YES
Q13885	YES
Q9R158	YES
Q86UR5	YES
Q96RW7	YES
O75369	YES
Q9UPN3	YES
Q9FZ06	YES
P07724	NO
P13639	YES
P49720	YES
Q6UB99	YES
Q8NEB7	YES

Table S4. *Cont.*

UniProt ID	The Site Is Deteced?
Q8WZ42	YES
Q9FVT2	YES
Q0WRR9	YES
P07954	YES
P06733	YES
P21333	YES
Q8WZ42	YES
Q9BYJ4	YES
P19171	YES
O75390	YES
Q14008	YES
Q33557	YES
Q43746	YES
Q6UB99	YES
P09622	YES
Q5VST9	YES
A2AUS0	YES
P10515	YES
O88307	YES
Q8WZ42	YES
P48347	YES
Q9UPN3	YES
P78527	YES
O75952	YES
P07288	YES
Q460N5	YES
Q9UBX3	NO
Q9FZ06	YES
Q92736	YES
Q14315	YES
Q6JEL2	YES
O75179	YES
Q8WXH0	YES
P98156	NO
Q8WZ42	YES
Q9NVA2	YES
P15924	YES
Q6JEL2	YES
Q8WXH0	YES
Q8WZ42	YES
Q9H7X3	YES
Q9XFH8	YES
Q15149	YES
Q92736	YES

Table S4. *Cont.*

UniProt ID	The Site Is Deteced?
Q460N5	YES
P13639	YES
P49454	YES
Q8NDX6	YES
Q14315	YES
Q9R158	YES
Q01813	YES
Q92736	YES
Q3TJ94	YES
Q9BS86	YES
Q8WZ42	YES
O49485	YES
P15924	YES
Q99798	YES
Q8IWV7	YES
P06733	YES
Q8WZ42	YES
Q96Q15	YES
P09972	YES
Q460N5	YES
P15104	YES
P60900	YES
Q9R158	YES
Q5VST9	YES
Q9BYJ4	YES
Q8WXH0	YES
P34791	YES
P78559	YES
Q12955	YES
Q99666	YES
Q14315	YES
Q9D8L4	YES
Q8WZ42	YES
Q92793	YES
Q9UPA5	YES
Q460N5	YES
Q8WXH0	YES
P00747	YES
Q9S7I3	YES
Q9WV42	NO
P35499	YES
P55072	YES
O03042	YES
Q99666	YES

Table S4. *Cont.*

UniProt ID	The Site Is Deteced?
P78527	YES
P02768	NO
P98156	NO
O75969	YES
Q8NEZ4	YES
Q06830	YES
Q8WZ42	YES
Q5VST9	YES
P12110	YES
Q6UB99	YES
P29511	YES
O95271	YES
Q6JEL2	NO
Q9NYC9	YES
Q9NYC9	NO
Q8WXH0	YES
Q13618	YES
P48047	YES
Q92736	YES
O14556	YES
Q6UVJ0	YES
O75969	YES
Q5TZA2	YES
Q01955	YES
Q9CWS0	YES
Q9D8L4	YES
Q96HH9	NO
Q92793	YES
Q6PKC3	YES
Q7Z4H7	YES
Q15149	YES
Q86UR5	YES
B1AXT2	YES
Q12955	YES
P45880	YES
O88307	NO
Q8NEZ4	YES
O75969	YES
P54609	YES
Q5VST9	YES
Q92793	YES
P42357	YES
Q8WZ42	YES
Q9Y6L6	YES

Table S4. *Cont.*

UniProt ID	The Site Is Deteced?
P37837	YES
Q96Q15	NO
P00747	YES
O43719	YES
P27140	YES
P15924	YES
P42357	YES
Q96RW7	YES
P08670	YES
Q8WZ42	YES
Q8WZ75	YES
O65396	YES
P54652	YES
Q9UI46	YES
Q9WV42	YES
Q3TJ94	YES
Q9R158	NO
Q96QE4	YES
P04075	YES
P10323	YES
Q8VY03	YES
P07205	YES
Q96RW7	YES
Q86UR5	YES
P35499	YES
P15924	YES
Q13011	YES
O75179	YES
P05091	YES
P98156	NO
Q8WXH0	YES
P49189	YES
P98064	YES
Q9NYC9	YES
O14715	YES
Q12955	YES
O94769	YES
P14152	YES
P78527	YES
Q99K10	YES
P00747	YES
P02768	YES
Q06830	YES
Q7Z4H7	YES

Table S4. *Cont.*

UniProt ID	The Site Is Deteced?
Q13535	YES
Q96RW7	YES
O15027	YES
P08752	YES
Q99996	YES
Q8TD31	YES
Q92736	YES
P98064	YES
Q8H0B7	YES
Q8WZ42	YES
Q96RW7	YES
Q96RW7	YES
Q8WZ42	YES
Q8WZ42	YES
Q9SGT4	YES
Q5VST9	YES
Q8WZ42	YES
Q8WYR1	YES
Q33557	NO
Q9WV42	YES
P27708	YES
P07195	YES
Q6Z8D9	YES
Q9SGT4	YES
Q92793	YES
P02768	YES
Q9WV42	NO
Q8WWI5	YES
P00747	YES
Q15149	YES
Q6UB99	YES
Q5VST9	NO
Q92736	YES
Q8IWV7	YES
Q92922	YES
Q9NYC9	YES
Q92736	YES
Q99666	YES
O95271	YES
Q9UPN3	YES
O75179	YES
Q8WZ42	YES
Q9SIB9	YES
Q9BXM0	YES

Table S4. *Cont.*

UniProt ID	The Site Is Deteced?
P27708	YES
Q5VST9	YES
Q9H8N7	YES
Q99666	YES
Q13939	YES
P68363	YES
Q96Q15	YES
Q8WZ42	YES
Q99666	YES
Q8WZ42	YES
Q9LR30	YES
Q9H7X3	YES
P12532	YES
Q8WZ42	YES
Q14990	YES
P26358	YES
P13639	YES
Q9NYC9	YES
Q8WZ42	YES
Q8WZ42	YES
Q8WZ42	YES
Q0WRR9	YES
O75369	YES
P98156	YES
Q14315	YES
Q16658	YES
O75037	YES
Q93008	YES
Q92736	YES
Q14524	YES
P49454	YES
Q547G3	YES
Q3LXA3	YES
P54265	NO
Q99996	YES
P07724	YES
Q12955	YES
O88307	NO
Q5VST9	YES
Q99KD4	YES
P12270	YES
P98064	YES
Q5VST9	YES
Q540M5	YES

Table S4. *Cont.*

UniProt ID	The Site Is Deteced?
P49189	YES
P34791	YES
Q5JQC9	YES
Q96ME7	YES
P26358	YES
P02788	YES
O88307	YES
Q8WYR1	YES
Q8WZ42	YES
Q8WXH0	YES
P08752	YES
P49327	YES
P40926	YES
Q9BYJ4	YES
P40939	YES
P19171	YES
P26358	YES
P04406	YES
Q9UPN3	YES
P00367	YES
B1AXT2	YES
Q9UQ13	YES
Q9NQ38	YES
P19367	YES
P02788	YES
Q8NDH3	YES
Q9Y2Q0	YES
P31040	YES
O08759	YES
P27612	YES
Q84WT8	NO
P42357	YES
Q9Y2Q0	YES
P49454	YES
P04075	YES
Q41088	YES
P35235	YES
Q8WZ42	YES
O75179	YES
Q5VST9	YES
P08238	YES
Q8IWV7	YES
Q9Y277	YES
P15924	YES
Q5VST9	YES
